# Thermo-Elastic Homogenization of TPMS-Based Interpenetrating Phase Composites: A Comparison Between Finite Element and Mechanics of Structure Genome Approaches

**DOI:** 10.3390/ma19214608

**Published:** 2026-10-30

**Authors:** Yao Koutsawa, Sara Mouman, Lucas Binsfeld, Levent Kirkayak, Jieun Yang, Joamin Gonzalez-Gutierrez, Gaetano Giunta

**Affiliations:** 1 Luxembourg Institute of Science and Technology (LIST), Structural Composites Research Unit, 5, rue Bommel, Hautcharage, L-4940 Luxembourg, Luxembourg; lucas.binsfeld@list.lu (L.B.); levent.kirkayak@list.lu (L.K.); joamin.gonzalez-gutierrez@list.lu (J.G.-G.); gaetano.giunta@list.lu (G.G.); 2 Faculty of Mechanical Engineering, Delft University of Technology, Mekelweg 2, 2628 CD Delft, The Netherlandsjieun.yang@tudelft.nl (J.Y.)

**Keywords:** interpenetrating phase composites, triply periodic minimal surfaces, thermo-elastic homogenization, mechanics of structure genome

## Abstract

Triply periodic minimal surface (TPMS)-based interpenetrating phase composites (IPCs) combine two co-continuous solid phases across a smooth, doubly curved interface, providing a multifunctional design space for tailoring mechanical and thermal properties. This study investigates the effective thermo-elastic and thermal-transport behavior of TPMS-based IPCs using two established homogenization approaches: finite element (FE)-based representative unit cell (RUC) homogenization and mechanics of structure genome (MSG)-based homogenization. Four TPMS topologies—gyroid, diamond (Schwarz D), PMY, and F-rhombic dodecahedron (F-RD)—are considered over TPMS-phase volume fractions ranging from 0.1 to 0.5. Conforming-mesh RUC models are generated using Microgen and analyzed in Abaqus for FE simulations with periodic boundary conditions, and in the in-house CmbsFE code for MSG-based homogenization. The two approaches are cross-verified for predicting effective elastic properties, coefficients of thermal expansion (CTEs), and thermal conductivities, and agree to within 0.3% on identical meshes. On identical meshes the two approaches solve linear systems of the same size, and MSG requires fewer matrix decompositions. The study further examines the influence of TPMS topology and TPMS-phase volume fraction on the multifunctional response of two-phase IPCs, supporting the design and optimization of structural–thermal materials.

## 1. Introduction

Lightweight, multifunctional materials are in growing demand across the aerospace, automotive, and energy sectors. Structural components in these sectors increasingly need high specific stiffness together with tailored thermal transport and dimensional stability. Examples are structural heat exchangers, battery thermal-management housings and precision optomechanical mounts. All of them must combine load-bearing capacity with controlled heat flow and minimal thermal distortion. Architected cellular materials and metamaterials have emerged as a design paradigm for meeting these coupled demands. Their effective properties depend not only on the constituent materials but also on a freely tunable unit-cell topology. This gives a much larger design space than conventional stochastic foams or fiber-reinforced composites offer [1,2,3]. However, realizing this potential requires predictive, physically grounded models. These models must resolve the coupled elastic and thermal response of such architectures. They must cover the topologies and volume fractions that can now be manufactured by additive manufacturing (AM).

Among architected topologies, those based on TPMS are a particularly promising class. Their smooth, doubly curved, non-self-intersecting interfaces minimize local stress and heat-flux concentrations relative to strut-based lattices. TPMS geometries were first identified mathematically by Schwarz [4] and Neovius [5] in the nineteenth century. Schoen [6] later classified these surfaces, establishing the gyroid, diamond, and further surfaces now in widespread engineering use. The same morphologies recur in biological and self-assembled systems, from butterfly-wing scales to block-copolymer mesophases. This suggests that evolutionary and thermodynamic optimization favor them as load- and transport-bearing interfaces [7]. The four topologies studied here are mathematical minimal surfaces. Their occurrence in nature and in engineering differs. The gyroid and the diamond (Schwarz D) surfaces are the most widespread, in nature and in additively manufactured heat exchangers, thermal-management devices and scaffolds [7,8,9,10]. The PMY and F-RD surfaces [6] are less common in applications. They can, however, be manufactured by additive manufacturing. All four topologies are therefore relevant to engineering and are not purely theoretical. TPMS RUCs are represented implicitly through trigonometric level-set functions. They can be instantiated as sheet or solid (network) architectures. Their TPMS-phase volume fraction can be tuned continuously. This gives a single geometric framework for a wide range of stiffness, energy-absorption and multifunctional design targets [8]. This tunability is not merely empirical. For the equal-volume-fraction Schwarz P and D surfaces, Jung and Torquato [11] showed that the bi-continuous morphology is near-optimal for fluid transport and for heat and electrical transport. By geometric analogy, it is also near-optimal for elastic stiffness under a compliant second phase. This provides a rigorous physical basis for TPMS interfaces as natural candidates for multifunctional composite design, rather than an arbitrary geometric choice.

This last observation motivates a natural evolution in TPMS design. Single-phase (sheet or solid) TPMS lattices leave one region void. TPMS-based IPCs instead fill both regions defined by the TPMS level set, with distinct, mutually continuous solid phases. In particulate or short-fiber composites, the reinforcing phase is discontinuous and load transfer relies on interfacial shear. An IPC is different: each phase is independently self-supporting and load-bearing, a topological feature already recognized in early micromechanical treatments of interpenetrating multiphase media [12]. When the TPMS surface itself defines the phase interface, this co-continuity yields smooth, low-concentration load paths between phases. It also improves damage tolerance through crack deflection and delocalization at the curved interface. It further gives access to property combinations that are difficult to realize with either constituent alone. Examples are simultaneously high stiffness and high toughness, or tailored stiffness–conductivity pairings [13,14]. Recent work has extended this concept toward increasingly application-relevant configurations. These include spinodal and stochastic TPMS-like topologies with tunable strength-toughness trade-offs [15]. They also include bicontinuous spinodal metamaterials. For these, the effective thermal conductivity, its directional dependence and its relation to the mechanical response have recently been studied by computational homogenization with periodic boundary conditions [16]. Spinodal and TPMS architectures are not interchangeable. The thermal-transport and thermo-mechanical questions addressed there are, however, closely related to those considered here. Beyond these, centimeter-scale carbon- and polymer-based architected IPCs have also been investigated. In the latter, matrix infiltration was shown to distribute stress effectively and delocalize failure, achieving specific energy-absorption levels competitive with wound fiber tubes [17]. A recent comprehensive review consolidates this rapidly growing body of work on TPMS-based IPCs, spanning structural, thermal, and even piezoelectric functionalities. It also highlights that quantitative, method-verified predictions of their coupled thermo-elastic and transport properties remain comparatively scarce [18].

Reliable exploitation of this design space requires efficient, accurate homogenization frameworks. These frameworks must predict the effective properties of a RUC as functions of topology and TPMS-phase volume fraction. The dominant benchmark approach is finite-element (FE) homogenization of the RUC under periodic boundary conditions (PBCs). It has been applied extensively to TPMS lattices to predict effective elastic moduli [19,20,21]. Complementary numerical strategies include voxel-based asymptotic homogenization [22] and Fast-Fourier-transform-based homogenization, the latter achieving comparable accuracy at substantially reduced computational cost for periodic microstructures [23]. FE-RUC homogenization is accurate and widely available. Its cost, however, grows quickly with the mesh size. This matters when several physics and many topology and volume-fraction combinations must be swept, as in the multifunctional design studies motivating this work.

The MSG offers an alternative route to the same effective properties. It was developed by Yu and co-workers from its predecessor, the Variational Asymptotic Method for Unit Cell Homogenization (VAMUCH) [24]. Our group has also applied and extended VAMUCH [25,26]. MSG casts homogenization as a single variational statement, solved once per physics on the structure genome (SG). For three-dimensionally periodic media, this recovers the same effective properties as RUC-based FE analysis, while requiring only inexpensive back-substitutions for each additional load case [27,28,29]. The theoretical equivalence and comparative accuracy of the two approaches were established for single-phase TPMS lattices in our companion study [30]. That work built on earlier applications of MSG to the effective elastic and thermal-conductivity properties of particulate and nano-reinforced composites [31,32]. Practical FE-RUC implementations, in turn, rely on established periodic-boundary-condition and finite-element-analysis frameworks for composite materials [33,34].

The elastic homogenization of TPMS architectures is now comparatively well established. Their thermal characterization is not. Thermal expansion and thermal conductivity have received considerably less systematic attention, although both are central to the multifunctional applications motivating this work. Existing studies of TPMS thermal conductivity show that effective conductivity is governed primarily by TPMS-phase volume fraction and topology. This has been shown for pure TPMS lattices manufactured by laser powder bed fusion [35], for TPMS-structured phase-change materials for battery thermal management [9] and for ceramic TPMS lattices [10]. It has also been shown for TPMS-based porous media more generally [36]. A smaller body of work considers two-phase TPMS foams and IPCs. There, the effective conductivity and the elastic moduli depend jointly on topology and phase contrast [37,38]. The underlying MSG-based thermal-conductivity homogenization framework has itself been established for composite materials with general interfacial conditions, including imperfect interfaces [32]. The CTE of TPMS-based IPCs is still markedly less explored. To our knowledge, only a handful of studies address it explicitly. The most notable is a numerical investigation of the combined thermo-elastic response of gyroid-based composites, including the role of phase-modulus contrast and Poisson’s ratio [39]. To the best of our knowledge, no study to date has jointly addressed effective elastic properties, CTE, and thermal conductivity for the same set of TPMS-based IPC topologies. Still less has any study compared FE-RUC and MSG predictions of all three property groups over a broad volume-fraction range.

This gap is significant. TPMS lattices, TPMS-based IPCs, FE-RUC homogenization, and the MSG/VAMUCH framework have each been well studied individually. Yet no systematic study has compared FE-RUC and MSG predictions of the effective elastic properties, CTE and thermal conductivity. This is missing for several TPMS-based IPC topologies over a broad range of TPMS-phase volume fractions. Closing this gap matters for two reasons. First, MSG requires a single factorization of the system matrix per physics, reused for all load cases. This is attractive for multi-physics and multi-configuration design studies. The present study compares the size of the linear systems, the number of matrix decompositions and the accuracy of the two approaches. Second, it is a prerequisite for understanding how topology and TPMS-phase volume fraction govern the coupled stiffness–CTE–conductivity trade-offs. These trade-offs are relevant to thermal-management and dimensionally stable structural applications.

To address this gap, we develop two-phase RUC models with conforming meshes for four TPMS-based IPC topologies: gyroid, diamond (Schwarz D), PMY, and F-rhombic dodecahedron (F-RD). TPMS-phase volume fractions from 0.1 to 0.5 are considered. For each configuration, we determine the effective elastic properties, coefficients of thermal expansion and thermal conductivities with two homogenization approaches applied to the same discretization. FE-RUC is run in Abaqus, with periodic boundary conditions imposed through the Micromechanics Plugin for Abaqus/CAE [33,40]. MSG/VAMUCH is run with the in-house CmbsFE implementation. We then assess the MSG predictions quantitatively against the FE-RUC results for all three groups of properties. The present work extends the purely elastic, single-phase comparison of our companion study [30] to the thermo-elastic and thermal-transport behaviour of two-phase IPCs. Neither homogenization method is new. FE-RUC homogenization with periodic boundary conditions and MSG/VAMUCH are well established, and their equivalence was examined in [30]. The contribution of this paper is the extension of that cross-verification to two-phase interpenetrating composites and to three coupled properties (elasticity, CTE and thermal conductivity). It also provides the resulting trends with topology and volume fraction. Finally, we examine the combined influence of topology and TPMS-phase volume fraction on stiffness, thermal expansion and conductivity. This identifies topology-dependent trade-offs for structures with coupled mechanical and thermal requirements. The geometries are generated with Microgen (Section 2.1). Python-based (Python 3.12.4) parametric CAD libraries such as CadQuery are also increasingly used to generate such three-dimensional structures [41,42].

The remainder of the paper is organized as follows. Section 2 describes the generation of the TPMS-based IPC RUCs and the FE- and MSG-based homogenization procedures. Section 3 presents and discusses the resulting effective properties, including the mesh and level-set convergence studies, the verification against the literature, the local fields, a quantitative comparison of the two homogenization approaches, and their computational cost. Section 4 presents concluding remarks.

## 2. Methodology

This section describes how the TPMS-based IPC representative unit cells are generated. It then gives the constituent phase properties. Finally, it presents the two homogenization strategies compared in this work: FE-based RUC homogenization and MSG-based homogenization. For each strategy, the effective elastic properties, CTEs and thermal conductivities are obtained with analogous, physically consistent procedures. The two approaches can thus be compared on an equal footing.

**Figure 1:**
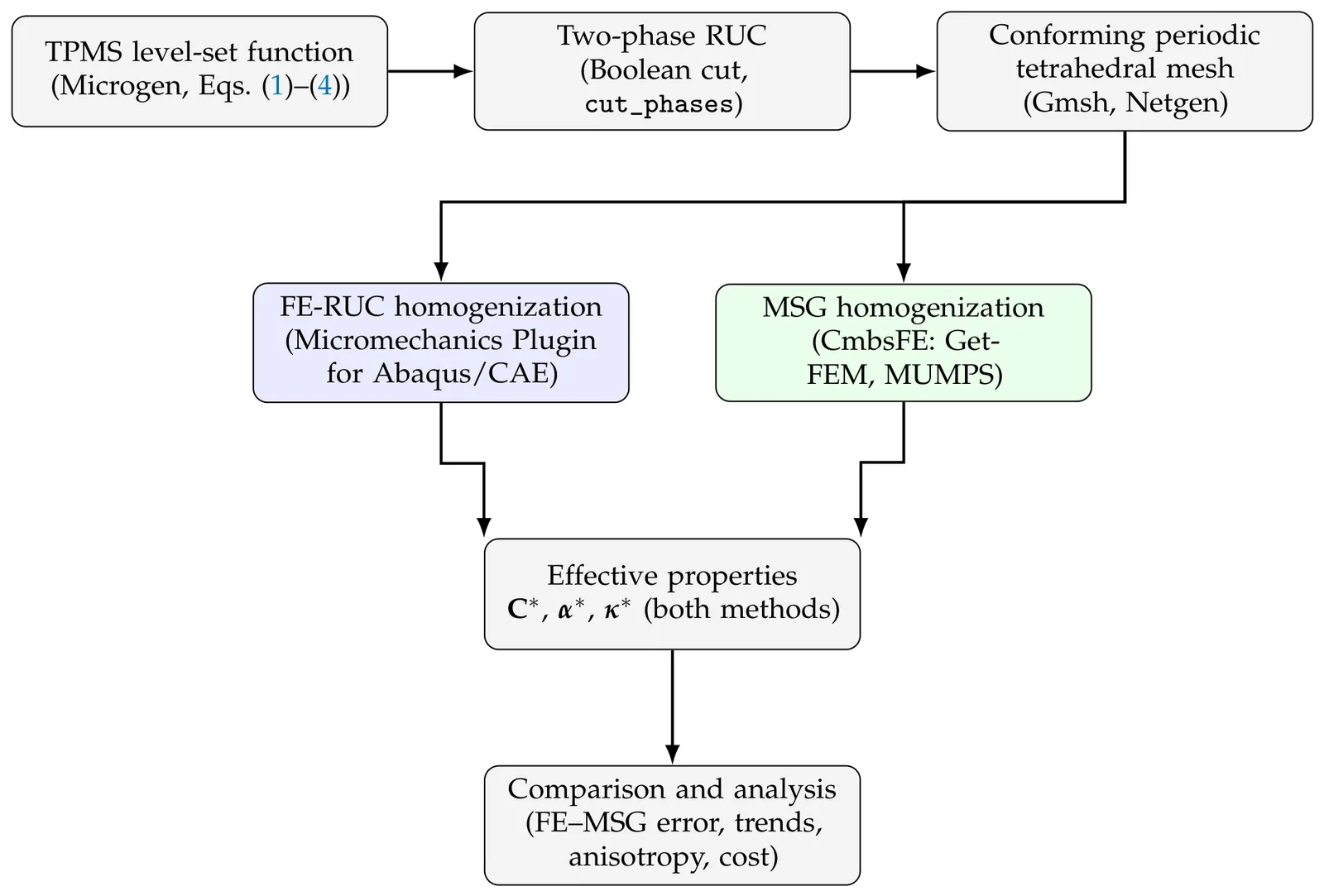
Workflow of the present study: geometry generation, meshing, homogenization with the two approaches on the same mesh, and comparison of the effective properties.

The overall procedure is summarized in Figure 1. A TPMS level-set function generates the two-phase RUC, which is meshed once with a conforming, periodic tetrahedral mesh. The same mesh is then homogenized with the FE-RUC approach (Abaqus Micromechanics Plugin) and with the MSG approach (CmbsFE). Both return the effective elasticity, CTEs and conductivity tensors, which are finally compared.

### 2.1. Generation of TPMS-Based Interpenetrating Phase Composite Unit Cells

The TPMS-based IPC microstructures are generated with the open-source Python library Microgen [43]. The modeling workflow is the same as in the companion study on single-phase TPMS architectures [30]. Four TPMS topologies are investigated, as in the companion study. They are the gyroid, the diamond (Schwarz D) surface, the PMY surface and the F-rhombic dodecahedron (F-RD, Schoen F-RD) surface. Each topology is implicitly defined, within a cubic cell, by a level-set function $\phi (x_{1},x_{2},x_{3})=0$. In Microgen, this function is expressed in the dimensionless coordinates $x_{i}=2\pi y_{i}/L$ (i=1,2,3). Here $y_{i}$ are the physical Cartesian coordinates of a point ***y*** in the cubic RUC of edge length *L*. This is the same local position vector ***y*** used to describe the RUC/SG domain throughout Section 2.3 and Section 2.4:

(1) $$\begin{array}{cc} \phi_{\mathrm{gyroid}}= & \sin x_{1}\cos x_{2}+\sin x_{2}\cos x_{3}+\sin x_{3}\cos x_{1},\; \end{array}$$

(2) $$\begin{array}{cc} \phi_{\mathrm{diamond}}= & \sin x_{1}\sin x_{2}\sin x_{3}+\sin x_{1}\cos x_{2}\cos x_{3}+\cos x_{1}\sin x_{2}\cos x_{3}+\\ \; & +\cos x_{1}\cos x_{2}\sin x_{3},\; \end{array}$$

(3) $$\begin{array}{cc} \phi_{\mathrm{PMY}}= & 2\cos x_{1}\cos x_{2}\cos x_{3}+\sin2x_{1}\sin x_{2}+\sin x_{1}\sin2x_{3}+\sin2x_{2}\sin x_{3},\; \end{array}$$

(4) $$\begin{array}{cc} \phi_{\mathrm{F-RD}}= & 4\cos x_{1}\cos x_{2}\cos x_{3}-\left(\cos2x_{1}\cos2x_{2}+\cos2x_{2}\cos2x_{3}+\cos2x_{3}\cos2x_{1}\right),\; \end{array}$$

each topology being generated, in the RUC, as the zero level set ϕ=0 of the corresponding function. A level-set sampling resolution of 25 was used for all RUCs, except for F-RD and PMY at ρ=0.1. For these two cases, a resolution of 40 was used. At low TPMS-phase volume fraction, the solid network is sensitive to under-resolution of the level-set sampling. At a resolution of 25, the generated F-RD mesh has a TPMS volume fraction of 0.487 instead of 0.1 (see Sensitivity to the level-set sampling resolution in Section 3.1). For PMY at ρ=0.1, the TPMS solid generated at this resolution fills the whole cell (volume fraction 1.0), so that no matrix phase remains. The finer resolution corrects both problems. In addition, F-RD has a strongly asymmetric level-set range, unlike the other three topologies.

The F-RD topology at ρ=0.1 required a second exception. With quadratic elements, its system has about $1.1\times 10^{6}$ unknowns for the thermo-elastic problem. This exceeded the memory (64 GB of RAM) available to the MUMPS direct solver of CmbsFE. The MSG analysis for this configuration therefore used linear (first-order) tetrahedral elements, rather than the quadratic elements used elsewhere in this study. For a fair comparison, the FE analysis for this same configuration was also run with linear elements. Every other topology–volume-fraction combination in this study uses quadratic elements for both FE and MSG. Section 3.8 discusses the FE–MSG agreement for this configuration.

Unlike the single-phase (sheet or solid) TPMS lattices considered previously [30], the microstructures investigated here are two-phase IPCs. For a given topology, the corresponding level-set function of Equations (1)–(4) partitions the cubic RUC of volume *V* into two fully interpenetrating, co-continuous solid phases. These two phases jointly fill the entire RUC volume, with no void space. All unit cells are cubes of edge length L=100 mm. The solid TPMS network (phase 1) is generated as a Microgen Tpms object with a prescribed TPMS-phase volume fraction $\rho =V_{1}/V$. Here $V_{1}$ is the volume enclosed by the TPMS solid region and *V* is the volume of the cubic RUC. The companion study [30] and Microgen (density parameter) call this quantity relative density. Since the two phases fill the RUC and no void is present, ρ is the volume fraction of the TPMS phase, and it is called so in this paper. It is varied from ρ=0.1 to 0.5 for each topology. The complementary phase (phase 2, denoted the “matrix” phase) occupies the remaining volume $V_{2}=V-V_{1}$, so that ρ+(1−ρ)=1. The two solid, conforming phases are obtained using the Boolean cutting procedure implemented in Microgen’s cut_phases function. This function subtracts the TPMS solid region from the cubic box. It returns two topologically complementary, non-overlapping solids. They share a common internal interface, which coincides with the TPMS level-set surface.

**Figure 2:**
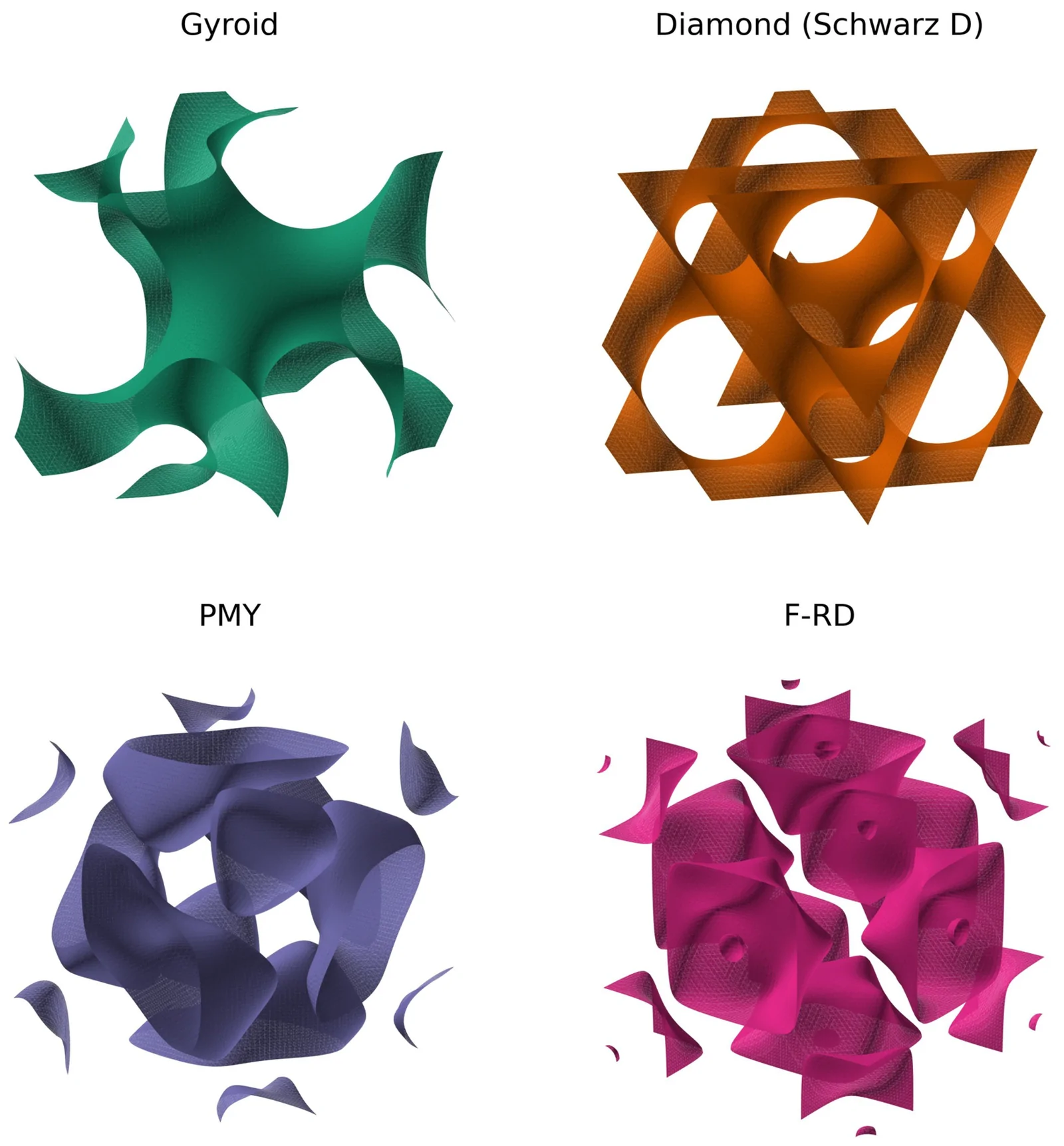
Solid TPMS network of the four topologies investigated (cubic unit cells of edge length L=100 mm), at TPMS-phase volume fraction ρ=0.3: gyroid, diamond (Schwarz D), PMY, and F-RD. Surfaces are extracted from the level-set functions of Equations (1)–(4) using a marching-cubes isosurface algorithm; colors match those used consistently for each topology in Section 3.

Figure 2 shows the solid TPMS network of the four topologies at ρ=0.3. The surfaces are extracted directly from the level-set functions of Equations (1)–(4). The color code (gyroid, diamond, PMY, F-RD) is used consistently for these four topologies throughout the remainder of this paper. In the two-phase IPC, the complementary matrix phase occupies the remaining volume of each unit cell. It shares the smooth, doubly curved interface visible in Figure 2.

**Figure 3:**
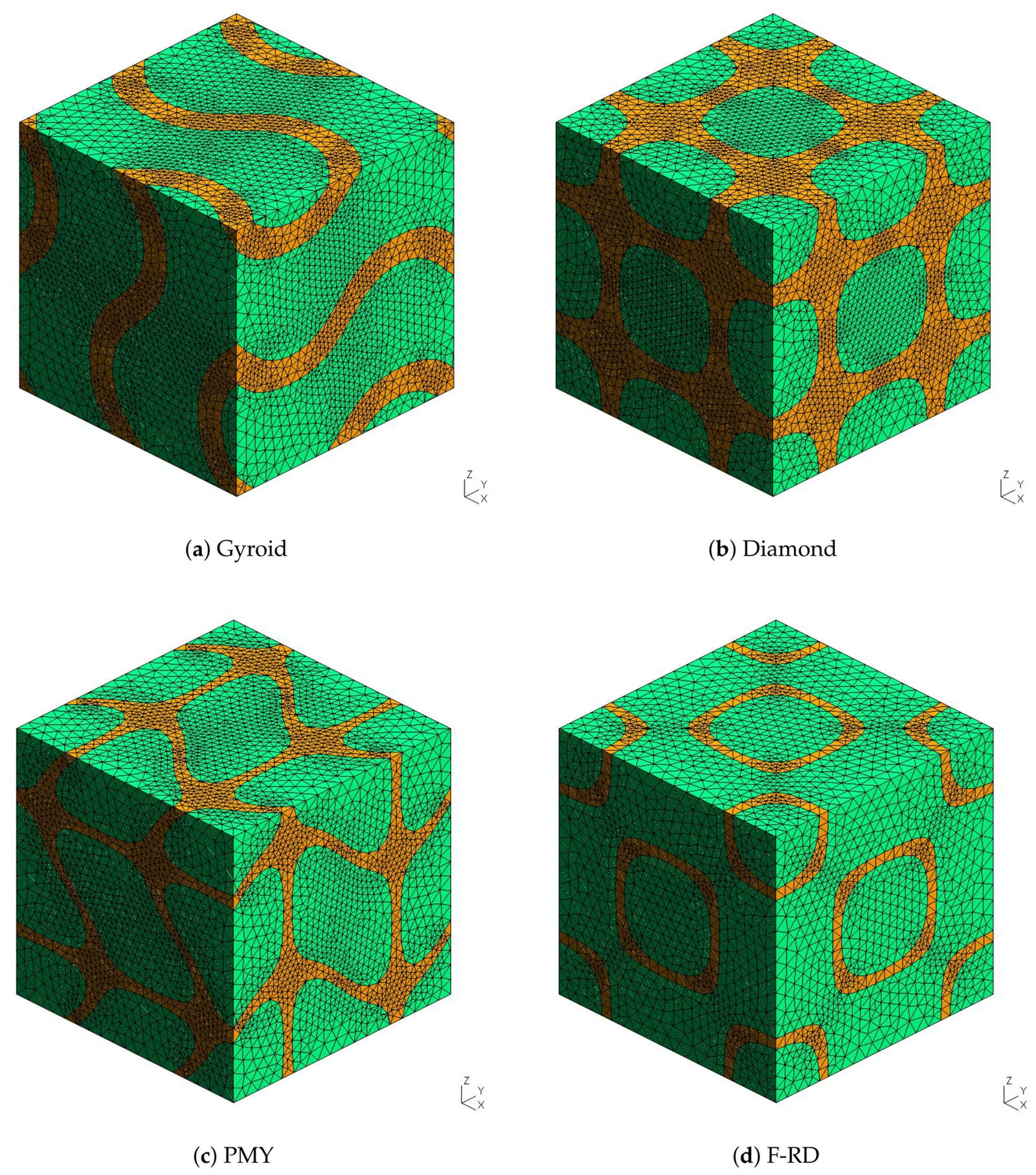
Conforming, periodic two-phase tetrahedral mesh of the four TPMS-based IPC topologies at TPMS-phase volume fraction ρ=0.3, generated with Gmsh (Section 2.1): (**a**) gyroid, (**b**) diamond, (**c**) PMY, (**d**) F-RD. The TPMS phase (orange) and matrix phase (green) share a conforming interface; this is the identical mesh used by both the FE (Section 2.3) and MSG (Section 2.4) homogenization analyses.

A conforming, periodic tetrahedral mesh of the two-phase RUC is subsequently generated with Gmsh [44] through Microgen’s mesh_periodic utility. The two-phase geometry is first imported into Gmsh via the OpenCASCADE kernel. The two phases are meshed as separate solids sharing a common interface, so a Boolean fragment operation is applied next. This operation shares (rather than duplicates) the interface faces, edges, and nodes between the two phases, ensuring a conforming mesh across the phase boundary. Periodicity is then enforced geometrically, prior to meshing. For each of the three RUC axes, the boundary faces on the negative side of the cube are paired with the corresponding faces on the positive side. The pairs are identified by matching bounding boxes offset by the RUC edge length. Each pair is declared periodic to Gmsh via an affine (translational) transformation. This constrains the mesher to generate identical node distributions on corresponding master and slave faces. The volume mesh is then generated for a prescribed target element size, using tetrahedral elements optimized with Netgen [45]. The element order is subsequently elevated to obtain quadratic (second-order) tetrahedral elements, consistent with the meshing strategy validated in the companion elastic study [30]. One exception applies, as noted above: F-RD at ρ=0.1 uses linear (first-order) elements for both FE and MSG. As a final safeguard, the periodicity of the generated mesh is verified numerically. For each pair of opposite faces, the in-plane coordinates of corresponding nodes are compared within a specified tolerance. The same periodic mesh is exported in two formats by a single geometry-and-meshing script. A Gmsh .msh file is used for the MSG analysis with CmbsFE (Section 2.4). An Abaqus .inp file is used for the FE analysis (Section 2.3). Both homogenization approaches therefore operate on an identical discretization of the RUC. Differences between the two sets of predictions cannot be attributed to geometry or mesh. Figure 3 shows the conforming, periodic two-phase meshes for the four TPMS-based IPC topologies at ρ=0.3. The mesh resolution adopted for the FE analyses is justified by the convergence study presented in Section 3.1.

### 2.2. Constituent Phase Properties

Both constituent phases are modeled as homogeneous, isotropic, linear thermo-elastic conducting solids. Each phase is characterized by five properties: a Young’s modulus *E*, a Poisson’s ratio ν, a coefficient of thermal expansion α, a thermal conductivity κ and a volumetric heat capacity $c_{v}$. Both phases are treated as generic engineering materials. They are distinguished only by their elastic-modulus, CTE, and thermal-conductivity contrasts, not as specific, named polymer, metal, or ceramic constituents. The elastic moduli, Poisson’s ratios, and CTEs follow the application example [46], itself based on the gyroid IPC studied by Chawla and Kiran [39]. Chawla and Kiran [39] report an elastic-modulus ratio of 20 between the two phases. On this basis, the COMSOL example [46] assumes 200 GPa for the TPMS phase and 10 GPa for the matrix phase, with a common Poisson’s ratio of 0.30. The corresponding CTEs are $0.8\times 10^{-6}$ 1/K (TPMS phase) and $44.0\times 10^{-6}$ 1/K (matrix phase). Neither [39] nor the COMSOL example [46] reports a thermal conductivity or specific heat capacity, since both are purely elastic/thermo-elastic studies. Representative thermal conductivities of 0.1 W/(m·K) (TPMS phase) and 0.3 W/(m·K) (matrix phase) are therefore assigned for the thermal-conductivity study. Both phases have a common volumetric heat capacity $c_{v}=1.65$ MJ/(m^3^·K). The heat capacity enters the energy expression of the CmbsFE/MSG formulation (Section 2.4.3) as the volumetric heat capacity $c_{v}$. No mass densities are assigned to the constituents, so $c_{v}$ is specified per unit volume. Only steady-state problems are considered here. The value of $c_{v}$ therefore does not influence any effective property reported in this work. The resulting properties are summarized in Table 1.

The two phases have different isotropic thermal conductivities, with a contrast ratio $\kappa_{\mathrm{matrix}}/\kappa_{\mathrm{TPMS}}=3$. The effective conductivity of the IPC reflects the geometric arrangement of the two phases (topology and TPMS-phase volume fraction) and this conductivity contrast. This is consistent with standard two-phase conductivity homogenization. The four unit cells have cubic symmetry, so the effective conductivity tensor is isotropic. The small directional differences found numerically are discretization effects (Section 3.3).

### 2.3. Finite-Element-Based Homogenization

The FE-based homogenization of the TPMS-based IPC RUCs is performed in Abaqus (version 2022), using the Micromechanics Plugin for Abaqus/CAE (version 1.19, 15 March 2024) [33,34,40]. It follows the general RUC homogenization framework verified for single-phase TPMS lattices in [30]. Here, this framework is extended to the coupled thermal-expansion and heat-conduction problems, using the numerical procedures described in [47].

**Figure 4:**
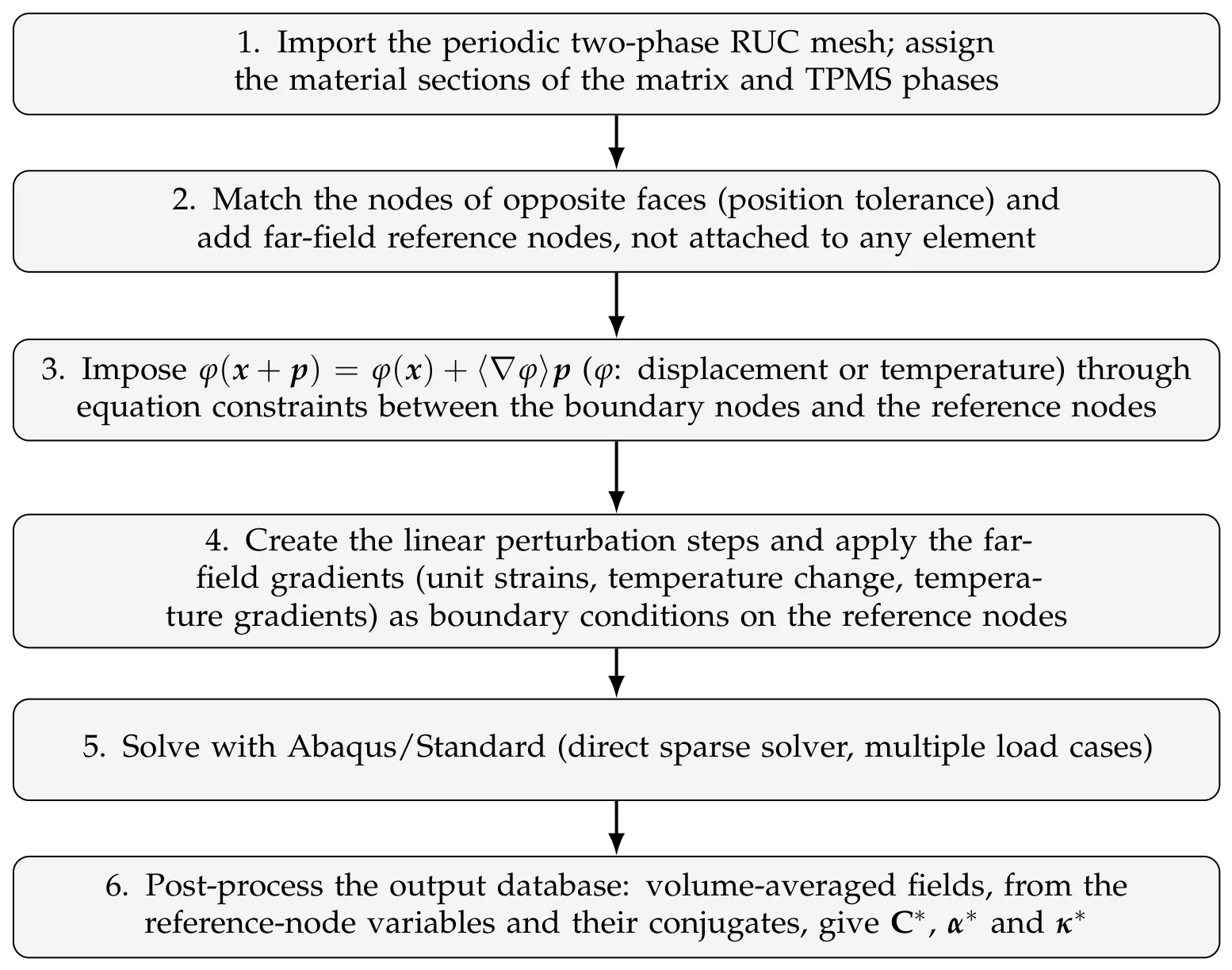
Block diagram of the homogenization procedure of the Micromechanics Plugin for Abaqus/CAE used for the FE-RUC analyses.

The sequence of operations carried out by the plugin [40] is summarized in Figure 4. The plugin matches the nodes of opposite faces and adds far-field reference nodes, which are not attached to any element. Periodicity is imposed by equation constraints between the boundary nodes and the reference nodes. The far-field gradients are applied as boundary conditions on the reference nodes.

#### 2.3.1. Periodic Boundary Conditions and Macro-Homogeneity

For a two-phase RUC of volume *V*, let $C_{ijkl}\left(y\right)$, $\alpha_{ij}\left(y\right)$ and $\kappa_{ij}\left(y\right)$ denote, respectively, the local (position-dependent) stiffness, CTE, and thermal-conductivity tensors at a point ***y*** within the RUC. Each phase is homogeneous and isotropic (Section 2.2). These piecewise-constant fields therefore take the matrix-phase properties of Table 1 in the matrix phase and the TPMS-phase properties in the TPMS phase. Throughout this section, a superposed asterisk (e.g., $C_{ijkl}^{*}$) denotes an effective (homogenized) property of the whole IPC. An overbar (e.g., ${\bar{\varepsilon }}_{ij}$) denotes a macroscopic (volume-averaged) field. Unmarked symbols (e.g., $\sigma_{ij}\left(y\right)$) denote local fields that vary from point to point within the RUC. The local displacement field $u_{i}$ at a point y within the RUC is decomposed into a macroscopic, affine part and a periodic fluctuation $\chi_{i}$ [30]:

(5) $$u_{i}\left(y\right)={\bar{\varepsilon }}_{ij}\;y_{j}+\chi_{i}\left(y\right),$$

where ${\bar{\varepsilon }}_{ij}$ is the prescribed macroscopic strain. Analogously, the local temperature field $\theta \left(y\right)=T\left(y\right)-T_{0}$ (with $T_{0}$ a stress-free reference temperature) is decomposed as

(6) $$\theta \left(y\right)=\bar{\nabla_{j}\theta }\;y_{j}+w\left(y\right),$$

where $\bar{\nabla_{j}\theta }$ is the prescribed macroscopic temperature gradient and *w* is a periodic temperature fluctuation. Periodic boundary conditions require that $\chi_{i}$ and *w* take equal values at corresponding points on opposite faces of the RUC [30,47], i.e.,

(7) $$\chi_{i}\left(y^{+}\right)=\chi_{i}\left(y^{-}\right),\;w\left(y^{+}\right)=w\left(y^{-}\right),$$

together with anti-periodicity of the corresponding tractions and normal heat fluxes on opposite faces. In Abaqus, Equation (7) is implemented through multi-point constraint equations. These link the degrees of freedom of matching node pairs on opposite RUC faces [30,33,47] (through far-field reference nodes in the Micromechanics Plugin for Abaqus/CAE, Figure 4). This is enabled by the conforming periodic mesh described in Section 2.1. PBCs are used throughout this study. Among the classical boundary-condition choices (kinematic-uniform, static-uniform and periodic), they are widely recognized as giving the most accurate estimate of the effective properties for a given RUC size. They also satisfy the Hill–Mandel macro-homogeneity condition of equivalent strain energy (respectively, thermal dissipation) between the heterogeneous RUC and its homogenized counterpart [30,47].

#### 2.3.2. Effective Elastic Properties in the FE-RUC Approach

The effective elastic response of the homogenized IPC is described by the generalized Hooke’s law, ${\bar{\sigma }}_{ij}=C_{ijkl}^{*}\;{\bar{\varepsilon }}_{kl}$, where ${\bar{\sigma }}_{ij}$ and ${\bar{\varepsilon }}_{ij}$ denote the volume-averaged (macroscopic) stress and strain,

(8) $${\bar{\sigma }}_{ij}=\langle \sigma_{ij}\rangle =\frac{1}{V}\int_{V}\sigma_{ij}\;dV,\;{\bar{\varepsilon }}_{ij}=\langle \varepsilon_{ij}\rangle =\frac{1}{V}\int_{V}\varepsilon_{ij}\;dV,$$

and $C_{ijkl}^{*}$ is the sought effective stiffness tensor of the IPC. Following [30], the full 6×6 effective stiffness matrix is populated column-by-column from six independent linear-elastic load cases. In each analysis, a single component of the macroscopic strain tensor is prescribed to unity through the PBCs of Equation (7), while all other components are set to zero. This gives three axial load cases (${\bar{\varepsilon }}_{11}$, ${\bar{\varepsilon }}_{22}$, ${\bar{\varepsilon }}_{33}$) and three shear load cases (${\bar{\gamma }}_{12}$, ${\bar{\gamma }}_{13}$, ${\bar{\gamma }}_{23}$). For each load case, the resulting local stress field $\sigma_{ij}\left(y\right)$, obtained from a static analysis of the two-phase RUC, is volume-averaged according to Equation (8). This yields the corresponding column of $C_{ijkl}^{*}$, expressed in contracted (Voigt) notation as the 6×6 matrix $C_{mn}^{*}$. The standard index pairs m,n=1,…,6 map the tensor indices as 11→1, 22→2, 33→3, 23→4, 13→5, 12→6. Only this direct, unit-strain extraction is used in the present study. The least-squares regression alternative of [47] is relevant for more general strain states and is not required here. The effective compliance matrix is obtained by inversion, $S_{mn}^{*}={\left[C_{mn}^{*}\right]}^{-1}$. The nine independent engineering constants of the (generally orthotropic) homogenized IPC are then recovered from the components of $S_{mn}^{*}$ [47]: the effective Young’s moduli $E_{1}^{*},E_{2}^{*},E_{3}^{*}$, shear moduli $G_{12}^{*},G_{13}^{*},G_{23}^{*}$, and Poisson’s ratios $\nu_{12}^{*},\nu_{13}^{*},\nu_{23}^{*}$.

#### 2.3.3. Effective Coefficients of Thermal Expansion in the FE-RUC Approach

The effective CTE tensor is obtained with the numerical procedure of [47]. It is based on the Duhamel–Neumann thermo-elastic constitutive law, which relates the macroscopic stress to the macroscopic total strain and a uniform temperature change ΔT:

(9) $${\bar{\sigma }}_{ij}=C_{ijkl}^{*}\left[{\bar{\varepsilon }}_{kl}-\alpha_{kl}^{*}\;\Delta T\right],$$

where $\alpha_{kl}^{*}$ is the sought effective CTE tensor. A single thermal-expansion analysis is performed. The PBCs of Equation (7) are applied with a zero prescribed macroscopic strain, ${\bar{\varepsilon }}_{kl}=0$, while a uniform temperature change ΔT is imposed over the entire RUC. Under these conditions, Equation (9) reduces, in contracted notation, to ${\bar{\sigma }}_{m}=-C_{nm}^{*}\;\alpha_{n}^{*}\;\Delta T$. The effective CTE vector is then recovered from the homogenized thermal stresses and the previously computed effective compliance matrix (Section 2.3.2) as

(10) $$\alpha_{n}^{*}=-\frac{1}{\Delta T}\;S_{nm}^{*}\;{\bar{\sigma }}_{m}.$$

#### 2.3.4. Effective Thermal Conductivity in the FE-RUC Approach

The effective thermal conductivity tensor is obtained from a steady-state heat-conduction analysis of the RUC. This is based on Fourier’s law, $q_{i}=-\kappa_{ij}\nabla_{j}\theta$, and the local energy balance $\nabla_{i}\left(\kappa_{ij}\nabla_{j}\theta \right)=0$ in the absence of internal heat generation [47]. Three thermal load cases are considered. The macroscopic temperature gradient is prescribed through the PBCs of Equation (7), along each of the three orthogonal directions in turn: ${\bar{\nabla_{j}\theta }}^{\;k}=\delta_{jk}\;\Theta$ (k=1,2,3), where Θ is the magnitude of the imposed temperature difference across the RUC. For each load case, the resulting local heat flux $q_{i}\left(y\right)$ and temperature gradient $\nabla_{i}\theta \left(y\right)$ fields are volume-averaged,

(11) $${\bar{q}}_{i}=\langle q_{i}\rangle =\frac{1}{V}\int_{V}q_{i}\;dV,\;\bar{\nabla_{i}\theta }=\langle \nabla_{i}\theta \rangle =\frac{1}{V}\int_{V}\nabla_{i}\theta \;dV.$$

The effective conductivity tensor $\kappa_{ij}^{*}$ is then obtained from the homogenized Fourier’s law, ${\bar{q}}_{i}^{\;k}=-\kappa_{ij}^{*}\;{\bar{\nabla_{j}\theta }}^{\;k}$, applied to the three load cases k=1,2,3 simultaneously:

(12) $$\kappa_{ij}^{*}=-\;{\bar{Q}}_{ik}{\left[{\bar{\nabla T}}^{-1}\right]}_{jk},\;{\bar{Q}}_{ik}\equiv {\bar{q}}_{i}^{\;k},\;{\bar{\nabla T}}_{jk}\equiv {\bar{\nabla_{j}\theta }}^{\;k}.$$

### 2.4. Mechanics of Structure Genome-Based Homogenization

The MSG-based homogenization is performed with CmbsFE, the in-house C++ implementation of the MSG framework. CmbsFE is built on GetFEM [48] (version 5.5) for finite element assembly, MUMPS [49,50] (version 5.9.0) for direct linear-system solution, and MuParser [51] for symbolic expression parsing. MUMPS is used with its OpenMP-parallel solver and an OpenMP-parallel BLAS library (OpenBLAS). The number of threads is set by the environment variable OMP_NUM_THREADS; with one thread, the solver runs serially. CmbsFE was previously used for the effective elastic properties of nano-composites and for the effective thermal conductivity of composites with imperfect interfaces [31,32]. It was extended to TPMS architectures in the companion elastic study [30]. MSG is a unified, semi-analytical multiscale constitutive theory [27,28]. It is based on the structure genome (SG) concept. The SG is the smallest mathematical building block of a material or structure that contains its complete constitutive information. For a material with three-dimensional heterogeneity, such as the TPMS-based IPCs, the SG coincides geometrically with the RUC of the FE analysis (Section 2.3). The same periodic mesh (Section 2.1) is used for both approaches [30].

#### 2.4.1. Variational Foundation

MSG constructs the homogenized (effective) model by minimizing, over the SG, the loss of information between the original heterogeneous body and the homogenized body. For a linear thermo-elastic conducting material, this loss is expressed in terms of strain energy and thermal “energy” [27,29,30]. The kinematic and thermal fields within the SG are decomposed, analogously to Equations (5) and (6), as

(13) $$u_{i}\left(x,y\right)={\bar{u}}_{i}\left(x\right)+\chi_{i}\left(x,y\right),\;\theta \left(x,y\right)=\bar{\theta }\left(x\right)+w\left(x,y\right).$$

Here, x and ***y*** are the macroscopic (structural-scale) and local (SG-scale) position vectors, respectively. They are distinct from the dimensionless level-set coordinates $x_{i}$ of Section 2.1. The vector ***y*** coincides with the local RUC position used in Section 2.3. ${\bar{u}}_{i}=\langle u_{i}\rangle$ and $\bar{\theta }=\langle \theta \rangle$ are the volume-averaged, homogenized displacement and temperature fields. $\chi_{i}$ and *w* are unknown, periodic fluctuation functions satisfying $\langle \chi_{i}\rangle =0$ and 〈w〉=0 [30,52]. The corresponding local strain and temperature-gradient fields are

(14) $$\varepsilon_{ij}={\bar{\varepsilon }}_{ij}+\chi_{\left(i,j\right)},\;\nabla_{i}\theta =\bar{\nabla_{i}\theta }+w_{,i},$$

where the comma denotes differentiation with respect to the local (micro) coordinates, and parentheses denote the symmetric part of the gradient.

#### 2.4.2. Effective Elastic Properties in the MSG Approach

For the purely mechanical problem, the strain-energy functional to be minimized over the SG, subject to the periodicity and zero-average constraints on $\chi_{i}$, is [29,30]

(15) $$\Pi =\langle \frac{1}{2}\;C_{ijkl}\left({\bar{\varepsilon }}_{ij}+\chi_{\left(i,j\right)}\right)\left({\bar{\varepsilon }}_{kl}+\chi_{\left(k,l\right)}\right)\rangle ,$$

whose Euler–Lagrange stationarity condition yields the governing equation for the fluctuation function,

(16) $${\left[C_{ijkl}\left({\bar{\varepsilon }}_{kl}+\chi_{k,l}\right)\right]}_{,j}=0,$$

with $\chi_{i}$ periodic on the SG boundary. Equation (16) is solved numerically via finite element discretization of the SG in CmbsFE. The solution is linear in the macroscopic strain, $\chi_{k}=H_{k}^{mn}\;{\bar{\varepsilon }}_{mn}$, where $H_{k}^{mn}$ is an influence-function tensor relating the macroscopic strains to the local displacement fluctuations. The effective stiffness tensor is then obtained by volume-averaging the strain-energy density [29,30]:

(17) $$C_{ijkl}^{*}=\langle C_{ijkl}+C_{ijmn}\;H_{\left(m,n\right)}^{kl}\rangle .$$

Here, $H_{\left(m,n\right)}^{kl}$ is the symmetric part of the gradient of $H_{m}^{kl}$ with respect to the local coordinate $y_{n}$. The comma-derivative and symmetrization convention is the same as for the local strain in Equation (14). The local strain and stress fields can subsequently be recovered by dehomogenization [29]:

(18) $$\varepsilon_{ij}={\bar{\varepsilon }}_{ij}+H_{\left(i,j\right)}^{mn}{\bar{\varepsilon }}_{mn},\;\sigma_{ij}=\left(C_{ijmn}+C_{ijkl}H_{\left(k,l\right)}^{mn}\right){\bar{\varepsilon }}_{mn}.$$

#### 2.4.3. Effective Coefficients of Thermal Expansion in the MSG Approach

For the coupled thermo-elastic problem, the strain-energy functional of Equation (15) is extended with the local thermal-stress coupling and thermal-storage terms. This follows the two-step MSG thermo-elastic homogenization of [52]

(19) $$\Pi =\langle \frac{1}{2}\;C_{ijkl}\varepsilon_{ij}\varepsilon_{kl}+b_{ij}\;\varepsilon_{ij}\;\theta +\frac{1}{2}\;c_{v}\;\frac{\theta^{2}}{T_{0}}\rangle ,$$

where $b_{ij}$ is the local thermal-stress coefficient tensor of a constituent phase, related to the local CTE and stiffness by $b_{ij}=-C_{ijkl}\alpha_{kl}$. The quantity $c_{v}$ is the local heat capacity per unit volume at constant volume (the product of the mass density and the mass-specific heat capacity). Since no mass densities are assigned to the constituents, $c_{v}$ is specified directly (Table 1). Discretizing the fluctuation functions $\chi_{i}$ and *w* over the SG mesh, and minimizing Equation (19), yields a linear system for the nodal fluctuation unknowns. This system is linear in both ${\bar{\varepsilon }}_{ij}$ and $\bar{\theta }$ [52]. Substituting the solution back into Equation (19) gives the homogenized strain-energy density in the reduced form

(20) $$\Pi =\frac{1}{2}\;{\bar{\varepsilon }}^{T}C^{*}\;\bar{\varepsilon }+{\bar{\varepsilon }}^{T}b^{*}\;\bar{\theta }+\frac{1}{2}\;c_{v}^{*}\;\frac{{\bar{\theta }}^{2}}{T_{0}},$$

where $C^{*}$ is the effective stiffness matrix of Equation (17), $b^{*}$ is the effective thermal-stress coefficient vector, and $c_{v}^{*}$ is the effective volumetric heat capacity. The effective CTE vector then follows directly as [52]

(21) $$\alpha^{*}=-{\left(C^{*}\right)}^{-1}b^{*}.$$

#### 2.4.4. Effective Thermal Conductivity in the MSG Approach

For the heat-conduction problem, MSG minimizes an analogous thermal “energy” functional over the SG [32,53]:

(22) $$\Phi =\langle \frac{1}{2}\left(\bar{\nabla_{i}\theta }+w_{,i}\right)\kappa_{ij}\left(\bar{\nabla_{j}\theta }+w_{,j}\right)\rangle ,$$

subject to 〈w〉=0 and periodicity of *w* on the SG boundary. The temperature fluctuation is discretized over the SG finite element mesh as

(23) w=SV,

where S is the matrix of standard finite element shape functions defined over the SG mesh, of the same type as used for the FE analysis of Section 2.3. V is the vector of unknown nodal values of the fluctuation function *w*. Imposing stationarity of Equation (22) with respect to V, subject to Equation (23), yields a linear system of the form $K\;V=-D_{hg}\;\bar{\nabla \theta }$. Here $K=\langle {\left(hS\right)}^{T}\kappa \left(hS\right)\rangle$ is the SG conductance matrix. $D_{hg}=\langle {\left(hS\right)}^{T}\kappa \rangle$ and $D_{gg}=\langle \kappa \rangle$ are further SG operator matrices, assembled from the local conductivity tensors κ of the two phases and the finite element shape functions of the SG mesh. h is the gradient (differential) operator matrix relating nodal shape-function values to their spatial derivatives over the SG [53]. The solution of this linear system is linear in the macroscopic temperature gradient, $V=V_{0}\;\bar{\nabla \theta }$, obtained from a single factorization of K. Substituting back into Equation (22) gives the reduced thermal energy $\Phi =\frac{1}{2}\;{\bar{\nabla \theta }}^{T}\kappa^{*}\;\bar{\nabla \theta }$, from which the effective thermal conductivity tensor is identified as [53]

(24) $$\kappa^{*}=V_{0}^{T}D_{hg}+D_{gg}.$$

The local temperature gradient and heat-flux fields within the SG can subsequently be recovered by dehomogenization [53]:

(25) $$\nabla_{i}\theta =\bar{\nabla_{i}\theta }+{\left(h\;S\;V_{0}\;\bar{\nabla \theta }\right)}_{i},\;q_{i}=-\kappa_{ij}\nabla_{j}\theta .$$

### 2.5. Comparison Framework Between the FE and MSG Approaches

Both approaches work on the same RUC/SG domain and the same periodic mesh (Section 2.1). Their governing equations are formally equivalent for a three-dimensional periodic heterogeneous medium [29]. With periodic boundary conditions, the FE boundary value problems of Section 2.3 and the MSG variational statements of Section 2.4 reduce to the same Euler–Lagrange equations. In the absence of numerical discretization and solver error, the two methods are therefore expected to yield identical effective properties and local fields. They differ, however, in computational implementation. Both approaches solve linear systems with several load cases (six unit strains and one temperature change for the thermo-elastic problem, three temperature gradients for the conductivity problem), but they differ in the number of matrix decompositions. In MSG the unknown is the periodic fluctuation, the coefficient matrix is the same for all load cases, and a single factorization serves all of them [29]. In an RVE analysis the number of decompositions depends on how the periodic constraints and the load cases are implemented. In the Micromechanics Plugin for Abaqus/CAE, the periodic boundary conditions are imposed on the total displacement by multi-point constraint equations of the form

(26) $$u_{i}\left(y^{+}\right)-u_{i}\left(y^{-}\right)-L\;{\bar{\varepsilon }}_{ij}=0.$$

The equations are written once for all pairs of nodes on opposite faces normal to $x_{j}$. Here *L* is the edge length of the RUC and ${\bar{\varepsilon }}_{ij}$ is a degree of freedom of a far-field reference node (Section 2.3.1). The coefficients of these equations (1, −1 and *L*) are the same for all load cases. The load cases differ only in the values prescribed to the reference-node degrees of freedom, that is, in the macroscopic strain. Partition the unknowns into free degrees of freedom $u_{f}$ and prescribed ones $u_{p}$. The system then reads $K_{ff}u_{f}=-K_{fp}u_{p}$. The prescribed values enter only the right-hand side. The matrix depends on the stiffness, on the coefficients of the constraint equations and on which degrees of freedom are prescribed. It is therefore identical for all load cases. Abaqus solves load cases that share the same matrix with a direct solver for multiple load cases. This solver performs a single matrix decomposition. In Abaqus, load cases are available only for linear perturbation procedures. The six unit-strain load cases are therefore defined as load cases of one linear perturbation step and solved with one decomposition and six right-hand sides. The temperature change of the CTE problem is a separate step with its own decomposition. The conductivity problem is solved with the steady-state heat-transfer procedure. Load cases are not available for this procedure. Each of the three temperature gradients is therefore a separate step with its own decomposition. The summary at the end of the Abaqus message files reports the passes through the equation solver that involve a matrix decomposition. It confirms two decompositions for a thermo-elastic job and three for a conductivity job. The MSG approach requires a single factorization of the SG system matrix for each physics (mechanical, thermo-mechanical or thermal). Inexpensive back-substitutions then solve the different load cases [29,30,53]. The numbers of decompositions are therefore two (FE) against one (MSG) for the thermo-elastic problem, and three against one for the conductivity problem. This feature of the formulation was already discussed for the purely elastic problem in [30]. The size of the linear systems, the number of matrix decompositions and the solver times of the two approaches are compared in Section 3.9.

## 3. Results and Discussion

This section first establishes the adequacy of the mesh resolution used in the numerical analyses (Section 3.1). The FE and MSG frameworks are then verified against previously published single-phase TPMS results (Section 3.2). Next, the effective elastic properties, coefficients of thermal expansion and thermal conductivities of the two-phase IPCs are presented property by property (Section 3.3, Section 3.4 and Section 3.5). A joint discussion of their multifunctional response follows (Section 3.6). Then come the local fields (Section 3.7), a quantitative comparison of the two homogenization approaches (Section 3.8) and their computational cost (Section 3.9).

### 3.1. Mesh Convergence Study

Before verifying the frameworks and presenting the two-phase IPC results, a mesh convergence study was performed for the gyroid topology. It covers the lowest and the highest TPMS-phase volume fractions, ρ=0.1 and ρ=0.5. The target element edge length $l_{e}$ was varied while the level-set sampling resolution used to generate the TPMS surface was kept fixed. For each mesh, the effective axial Young’s modulus $E_{1}^{*}$, coefficient of thermal expansion $\alpha_{1}^{*}$, and thermal conductivity $\kappa_{1}^{*}$ were evaluated using the FE-based RUC procedure described in Section 2.3.

Table 2 and Table 3 show that the predicted properties converge rapidly at both TPMS-phase volume fractions. For ρ=0.1, reducing the target element edge length from 10 mm to 3 mm changes $E_{1}^{*}$, $\alpha_{1}^{*}$, and $\kappa_{1}^{*}$ by less than 0.05%, 0.04%, and 0.01%, respectively. The corresponding changes at ρ=0.5 are below 0.21%, 0.09%, and 0.02%, respectively.

Based on these results, a target element edge length of $l_{e}=6$ mm was selected for the analyses reported in this study. For a 100 mm unit cell, this corresponds to approximately 94,300 elements at ρ=0.1 and 88,100 elements at ρ=0.5. The selected mesh agrees with the finest mesh to within 0.14% for all three effective properties at both TPMS-phase volume fractions. Its computational cost is suitable for the full set of topology–volume-fraction configurations.

The mesh study comprises two further parts. First, the FE–MSG difference was tracked along a mesh-refinement sequence. The sequence covers the gyroid (ρ=0.5, four levels) and the more demanding PMY topology (ρ=0.5, three levels). Both methods were run on identical quadratic-tetrahedron meshes (Table 4). Second, the sensitivity to the level-set sampling resolution was examined for the F-RD topology at ρ=0.1 (Table 5).

Table 4 shows that, on the same mesh, the two formulations agree to within 0.05% for $E_{1}^{*}$ and for $\alpha_{1}^{*}$ at every refinement level. The conductivity $\kappa_{1}^{*}$ is identical to the three decimals reported. The FE–MSG difference does not grow with refinement; it is largest on the coarsest gyroid mesh, and it is 0.02% or less for $l_{e}\leq 6$ mm and for PMY. It is therefore a discretization-level effect and not a systematic formulation difference. Both methods also converge to the same limit. For the gyroid, $E_{1}^{*}$ changes between $l_{e}=10$ mm and $l_{e}=3$ mm by 0.20% (FE) and 0.16% (MSG). For PMY, it changes by 0.42% between $l_{e}=9$ mm and $l_{e}=4$ mm for both methods.

With a sampling resolution of 25, the generated F-RD mesh has a TPMS volume fraction of 0.487 instead of 0.1. The effective properties are then meaningless for the intended configuration ($E_{1}^{*}\approx 58$ GPa against about 20 GPa). Resolutions 40 and 60 both reproduce the target volume fraction to within 0.15%. Moving from 40 to 60 changes $E_{1}^{*}$ by −1.1%, $\alpha_{1}^{*}$ by +0.5% and $\kappa_{1}^{*}$ by +0.08% (FE). The resolution of 40 used for this case is therefore adequate for the conclusions drawn. The uncertainty on the stiffness is about 1%. The FE–MSG difference at resolution 40 is −0.22% for $E_{1}^{*}$, +0.28% for $\alpha_{1}^{*}$ and +0.10% for $\kappa_{1}^{*}$.

### 3.2. Verification of the FE and MSG Frameworks Against Literature Results

Before presenting the new two-phase IPC results, the FE and MSG homogenization procedures of Section 2 are verified against two previously published reference cases. The same RUC/SG generation, meshing and solution workflow is used here for single-phase lattices (bulk-material limit) of the gyroid and diamond topologies. This follows the verification strategy of the companion elastic study [30]. The two reference cases are the titanium gyroid lattice of Refai et al. [19] (ρ=0.1, bulk properties $E_{s}=110$ GPa, $G_{s}=41$ GPa, $\nu_{s}=0.34$) and the diamond lattice of Zhang et al. [21] (ρ=0.3, bulk properties $E_{s}=190$ GPa, $G_{s}=73$ GPa, $\nu_{s}=0.30$). The latter includes a numerical (FEM) result and two experimental (EXP) measurements. Table 6 compares the effective properties obtained in this study with these literature values.

The two lattices were generated with Microgen 1.3.2 (single solid phase, periodic quadratic tetrahedral meshes, level-set sampling 50, element size 0.1 mm for the gyroid and 0.2 mm for the diamond), and both methods were run on the same mesh. On these meshes the MSG and FE Young’s moduli differ by 0.37% (gyroid) and 0.07% (diamond). These differences are larger than for the two-phase IPCs (Section 3.8). They are small compared with the deviations from the literature values. Both the FE and MSG predictions agree with the gyroid reference of Refai et al. [19] to within 1.9%, and with the diamond FEM reference of Zhang et al. [21] to within 0.1%. The largest deviation, 4.53%, is against the first experimental measurement of Zhang et al. It is consistent with the numerical-experimental scatter reported by the original authors. These deviations are consistent with, and in fact tighter than, those reported for the same verification cases in the companion elastic study [30]. This confirms that the FE and MSG frameworks reproduce established literature results. They can therefore be applied to the new two-phase IPC configurations discussed below. These cases concern the elastic properties of single-phase TPMS lattices. They verify the mechanical part of the two frameworks. They do not validate the thermal-expansion and thermal-conductivity predictions for the two-phase IPCs, for which no experimental reference is available here. The agreement between FE and MSG in Section 3.8 is likewise a cross-verification of two numerical formulations that are expected to solve the same problem. It is not a physical validation.

### 3.3. Effective Elastic Properties

**Figure 5:**
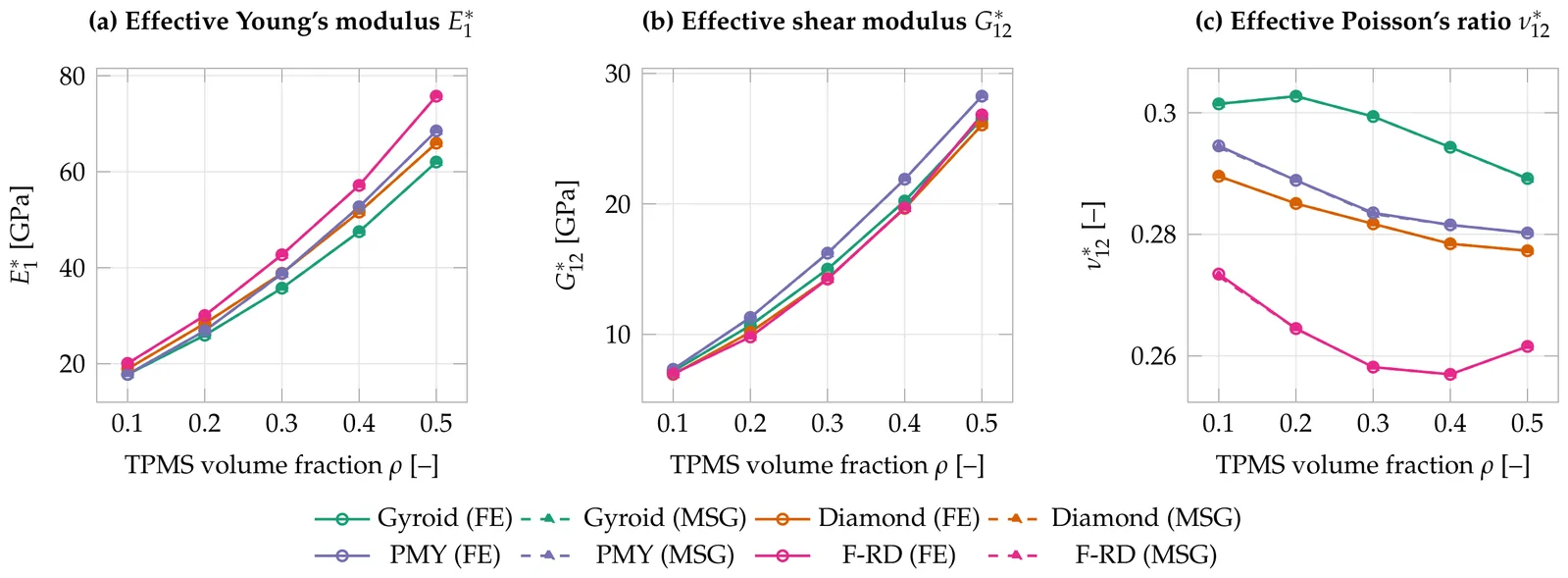
Effective elastic properties of the four TPMS-based IPC topologies as a function of TPMS-phase volume fraction ρ: (**a**) Young’s modulus $E_{1}^{*}$, (**b**) shear modulus $G_{12}^{*}$, (**c**) Poisson’s ratio $\nu_{12}^{*}$.

Figure 5 presents the effective axial Young’s modulus $E_{1}^{*}$, shear modulus $G_{12}^{*}$ and Poisson’s ratio $\nu_{12}^{*}$ of the four topologies. They are given as functions of the TPMS-phase volume fraction ρ, as predicted by the FE and MSG approaches.

Table 7 reports the numerical values underlying Figure 5, for all four topologies and the full volume-fraction range investigated.

For all four topologies, $E_{1}^{*}$ and $G_{12}^{*}$ increase monotonically with TPMS-phase volume fraction. At ρ=0.1, $E_{1}^{*}$ ranges from about 17.8 to 20.1 GPa across the four topologies. At ρ=0.5, it ranges from about 62 to 76 GPa. This increase is consistent with the growing volume fraction of the stiffer TPMS phase (E=200 GPa), relative to the compliant matrix phase (E=10 GPa, Table 1). The F-RD topology is the stiffest at every TPMS-phase volume fraction investigated. At ρ=0.1, F-RD leads by about 13% over the softest topologies, gyroid and PMY. This gap widens with TPMS-phase volume fraction. At ρ=0.5, F-RD leads by about 22% over the gyroid and by about 11% over PMY. The gyroid topology is consistently the most compliant. Diamond and PMY fall between gyroid and F-RD, with their relative ranking varying with ρ. The effective Poisson’s ratio tends to decrease with increasing TPMS-phase volume fraction, from $\nu_{12}^{*}\approx 0.27$–0.30 at ρ=0.1 towards $\nu_{12}^{*}\approx 0.26$–0.29 at ρ=0.5. The decrease is not strictly monotonic: the gyroid value increases slightly between ρ=0.1 and ρ=0.2, and F-RD reaches a minimum at ρ=0.4. This reflects the progressively smaller influence of the compliant, higher-Poisson’s-ratio matrix phase, as the stiffer TPMS network comes to dominate the load path. The same qualitative trend was reported for single-phase TPMS lattices in the companion study [30].

Only the axial components $E_{1}^{*}$, $G_{12}^{*}$ and $\nu_{12}^{*}$ are shown in Figure 5. Across all four topologies, all five TPMS-phase volume fractions, and both homogenization methods, the remaining tensor components differ from their axial counterparts by at most 0.07% (Young’s modulus), 0.05% (shear modulus), and 0.14% (Poisson’s ratio). The near-equality of the axial and shear components shows that the effective elasticity tensor has cubic symmetry, as expected from the point-group symmetry of the unit cells. Cubic symmetry is not isotropy, however: a cubic material has three independent elastic constants, and isotropy requires the additional relation $2C_{44}=C_{11}-C_{12}$. The degree of elastic anisotropy is therefore quantified by the Zener ratio $A=2C_{44}/\left(C_{11}-C_{12}\right)$, with A=1 for an isotropic material (Table 8). The values range from 0.83 to 1.10. For the gyroid and PMY composites, A>1: the shear stiffness $C_{44}$ exceeds the value $(C_{11}-C_{12})/2$ of an isotropic material with the same $C_{11}$ and $C_{12}$. For F-RD, and for diamond at low volume fraction, A<1. F-RD is the most anisotropic topology, with *A* as low as 0.83, i.e., a deviation of about 17.5% from isotropy. The IPCs studied here should therefore be described as cubic and not as quasi-isotropic. The axial values $E_{1}^{*}$, $G_{12}^{*}$ and $\nu_{12}^{*}$ of Figure 5 do not characterize the elastic response by themselves.

**Figure 6:**
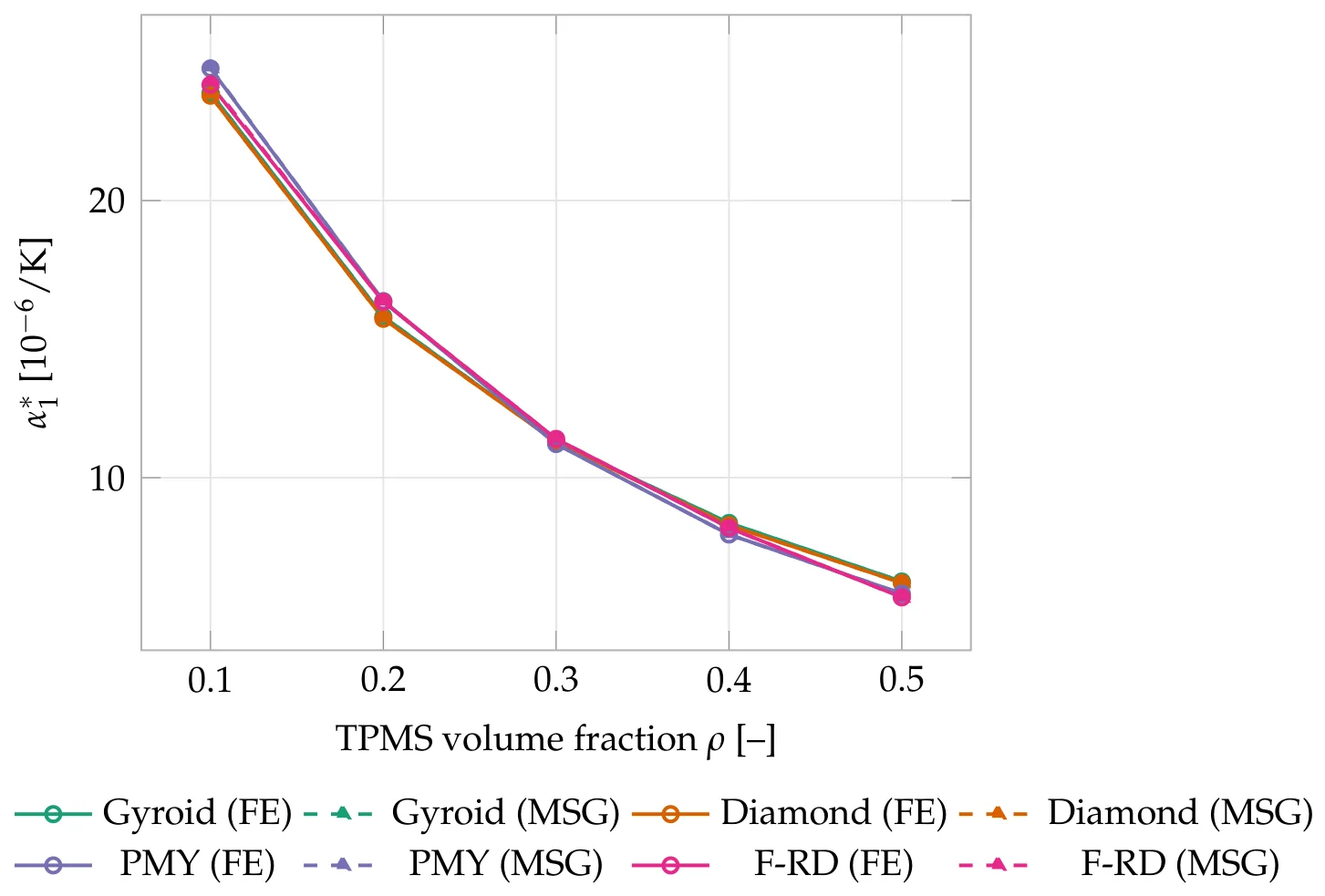
Effective axial coefficient of thermal expansion $\alpha_{1}^{*}$ of the four TPMS-based IPC topologies as a function of TPMS-phase volume fraction ρ.

**Figure 7:**
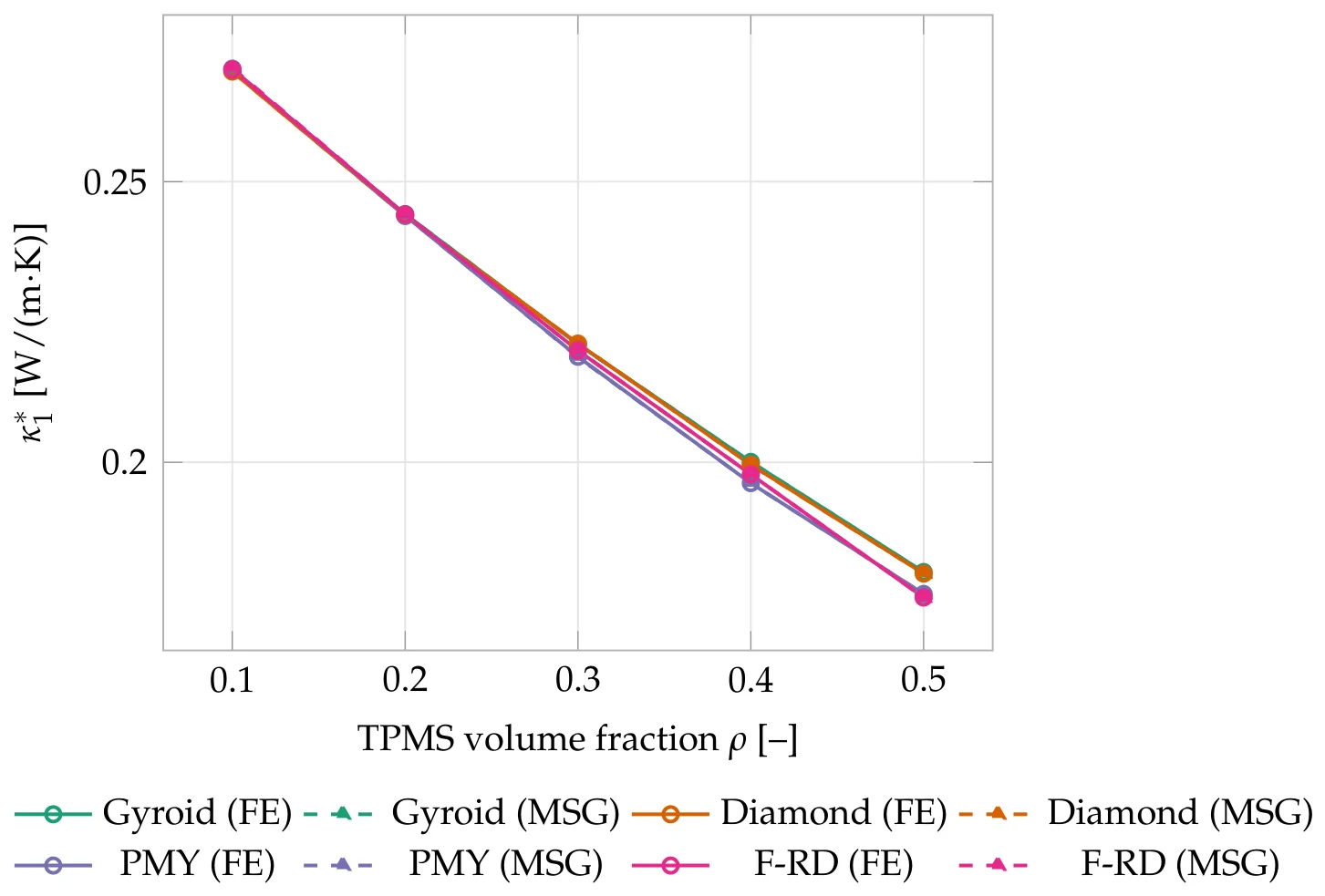
Effective axial thermal conductivity $\kappa_{1}^{*}$ of the four TPMS-based IPC topologies as a function of TPMS-phase volume fraction ρ.

For second-order tensors, cubic symmetry implies isotropic behaviour. Any difference between the three diagonal components, or any non-zero off-diagonal component, is therefore a numerical artefact of the discretization. Over all topologies and volume fractions, the three diagonal components of the CTE tensor differ by at most 0.14%. Those of the conductivity tensor differ by at most 0.012%. The off-diagonal components do not exceed 0.6% (CTE) and 0.024% (conductivity) of the mean diagonal value. The axial values $\alpha_{1}^{*}$ and $\kappa_{1}^{*}$ reported in Figure 6 and Figure 7 are therefore representative of the full tensors.

### 3.4. Effective Coefficients of Thermal Expansion

Figure 6 presents the effective axial CTE $\alpha_{1}^{*}$ as a function of TPMS-phase volume fraction.

Table 9 reports the numerical values underlying Figure 6, for all four topologies and the full volume-fraction range investigated.

The effective CTE decreases monotonically with increasing TPMS-phase volume fraction for all four topologies. At ρ=0.1, $\alpha_{1}^{*}$ ranges from about 23.8 to $24.8\times 10^{-6}$/K across the four topologies. At ρ=0.5, it ranges from about 5.7 to $6.3\times 10^{-6}$/K. This decrease occurs because the low-CTE TPMS phase ($\alpha =0.8\times 10^{-6}$/K) increasingly constrains the thermal expansion of the high-CTE matrix phase ($\alpha =44\times 10^{-6}$/K). The reduction is markedly stronger than a rule-of-mixtures estimate would suggest. For example, at ρ=0.1, a Voigt-type volume average gives $\alpha \approx 0.1\times 0.8+0.9\times 44\approx 39.7\times 10^{-6}$/K, roughly 1.6–1.7× the values in Figure 6. This reflects the elastic-constraint (Turner-type) mechanism of the Duhamel–Neumann formulation of Section 2.3.3. The stiffer TPMS network suppresses the free thermal expansion of the compliant matrix, well beyond a volume-weighted average. Unlike the elastic properties, no single topology has the lowest CTE across the whole volume-fraction range. Diamond has the lowest CTE at ρ=0.1 and ρ=0.2. PMY takes over at ρ=0.3 and ρ=0.4. F-RD has the lowest CTE only at ρ=0.5, where it is also the stiffest topology (Section 3.3). This shifting ranking shows that the CTE–stiffness relationship is not a simple, universal trend across topologies. It depends on the specific network geometry at each TPMS-phase volume fraction.

### 3.5. Effective Thermal Conductivity

Figure 7 presents the effective axial thermal conductivity $\kappa_{1}^{*}$ as a function of TPMS-phase volume fraction.

Table 10 reports the numerical values underlying Figure 7, for all four topologies and the full volume-fraction range investigated.

The effective conductivity decreases monotonically with TPMS-phase volume fraction for all four topologies. At ρ=0.1, $\kappa_{1}^{*}$ ranges from about 0.2696 to 0.2701 W/(m·K) across the four topologies: a narrow spread of less than 0.2%. At ρ=0.5, the spread widens to about 0.176–0.180 W/(m·K). This decrease occurs as the low-conductivity TPMS phase (κ=0.1 W/(m·K)) progressively displaces the higher-conductivity matrix phase (κ=0.3 W/(m·K)). At ρ=0.1, the predicted values for all four topologies fall within the Hashin–Shtrikman bounds for a two-phase isotropic conductor of the same constituent conductivities and volume fractions ($\kappa_{\mathrm{HS}}^{-}=0.269$, $\kappa_{\mathrm{HS}}^{+}=0.275$ W/(m·K)). This provides an additional, physics-based consistency check (a bound, not a validation) on the conductivity homogenization procedure, beyond the mesh convergence study of Table 2. As with the CTE, no single topology has the lowest conductivity across the whole volume-fraction range. Diamond is lowest at ρ=0.1–0.2; PMY is lowest at ρ=0.3 and 0.4; F-RD is lowest only at ρ=0.5, mirroring the CTE ranking of Section 3.4.

### 3.6. Cross-Property Discussion

Figure 5, Figure 6 and Figure 7 reveal a consistent multifunctional trade-off with TPMS-phase volume fraction. Increasing TPMS-phase volume fraction raises stiffness. At the same time, it lowers both CTE and thermal conductivity. All three effective properties are governed by the same underlying competition: the stiff, low-CTE, low-conductivity TPMS phase against the compliant, high-CTE, higher-conductivity matrix phase. The four topologies respond to this trade-off differently. Stiffness ranking is stable with volume fraction: gyroid is the most compliant topology, and F-RD is the stiffest, at every TPMS-phase volume fraction investigated. CTE and thermal conductivity rankings are not stable. Diamond has the lowest CTE and conductivity at low TPMS-phase volume fraction. PMY takes over at intermediate volume fractions. F-RD only becomes the lowest-CTE, lowest-conductivity topology at ρ=0.5, where it is also the stiffest. At that volume fraction, F-RD combines the best stiffness, CTE, and conductivity of the four topologies simultaneously. At lower volume fractions, the stiffest topology and the best thermal-transport topology are not the same. From a design standpoint, this has a direct implication. At low TPMS-phase volume fraction, a designer should choose diamond for the lowest thermal expansion and conductivity, or F-RD for the highest stiffness. A single topology cannot deliver both. At high TPMS-phase volume fraction, F-RD is the clear choice: it dominates all three properties at once for the constituent properties of Table 1. The gyroid topology has a low stiffness and a comparatively strong thermal transport at high volume fraction. It remains preferable where thermal conductivity is the priority, such as in heat-exchanger applications.

### 3.7. Local Fields

**Figure 8:**
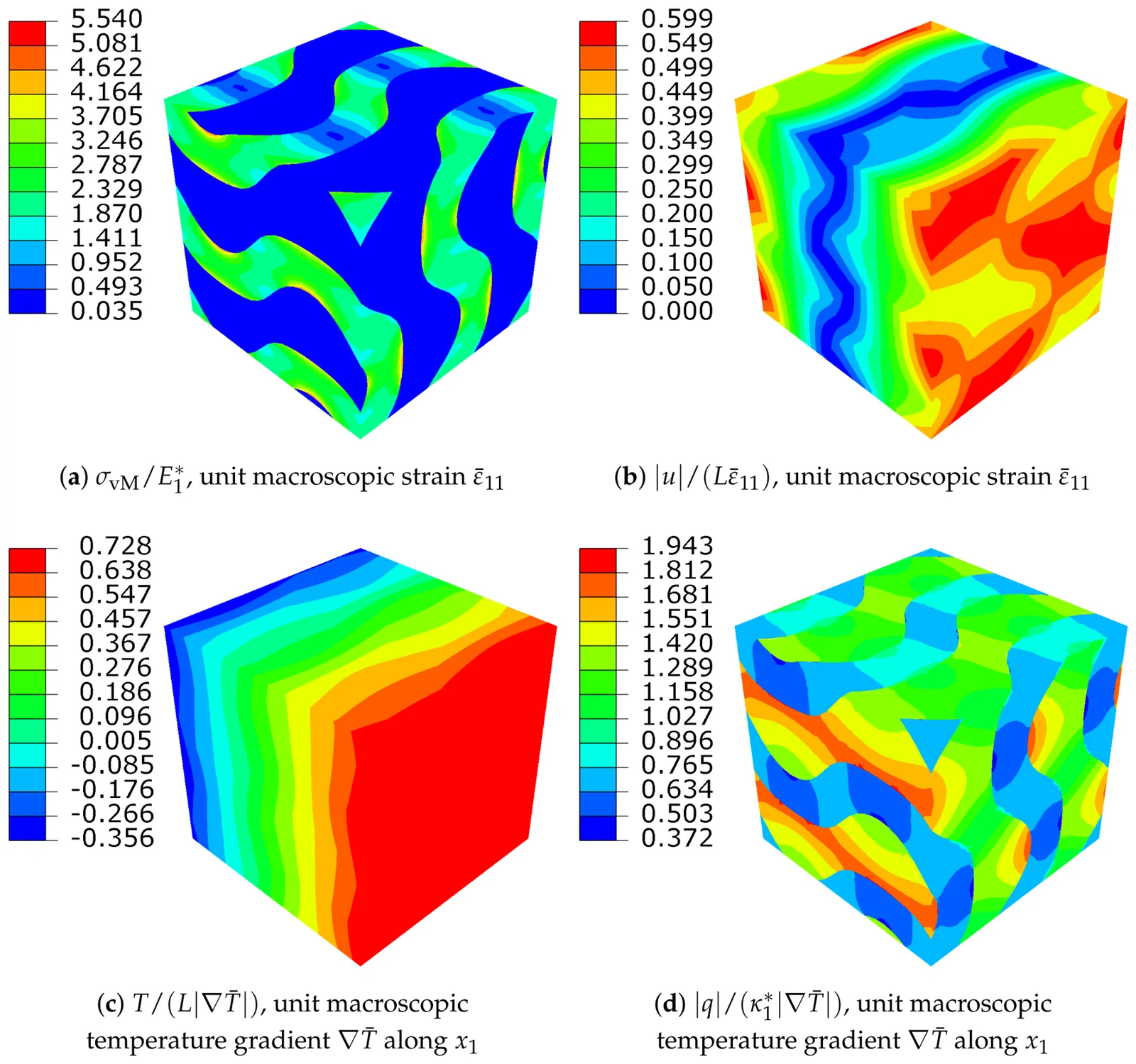
Local fields in the gyroid IPC at ρ=0.5 ($l_{e}=6$ mm, quadratic tetrahedra) from the FE-RUC analysis, for unit macroscopic loads: (**a**) normalized von Mises stress and (**b**) normalized displacement magnitude (deformed shape, scaled) for a unit macroscopic strain ${\bar{\varepsilon }}_{11}$; (**c**) normalized temperature and (**d**) normalized heat-flux magnitude for a unit macroscopic temperature gradient $\nabla \bar{T}$ along $x_{1}$. All fields are dimensionless; the normalizations are given in the sub-captions.

Figure 8 shows the local fields of the FE-RUC analysis for the gyroid IPC at ρ=0.5. The loads are the unit macroscopic loads that define the effective properties: a unit macroscopic strain ${\bar{\varepsilon }}_{11}$ for the mechanical problem and a unit macroscopic temperature gradient $\nabla \bar{T}$ along $x_{1}$ for the thermal problem. All fields are shown in dimensionless form. The stress is normalized by the effective Young’s modulus $E_{1}^{*}$, the displacement by $L{\bar{\varepsilon }}_{11}$, the temperature by $L|\nabla \bar{T}|$ and the heat flux by $\kappa_{1}^{*}\left|\nabla \bar{T}\right|$. Here *L* is the edge length of the RUC. Under the macroscopic strain, the stiff TPMS phase carries most of the stress. The stress level in the compliant matrix is markedly lower, in line with the modulus ratio of 20 between the phases (Figure 8a). The displacement field is smooth and periodic (Figure 8b). For the thermal problem, the temperature varies almost linearly across the cell. The periodic fluctuation induced by the conductivity contrast is small (Figure 8c). The heat flux is distributed non-uniformly over the two phases (Figure 8d). These fields illustrate the local response from which the effective properties of the previous sections are obtained by volume averaging. The fields are reported for unit macroscopic loads and a single configuration; the effective properties, and not the local fields, are the object of this study.

### 3.8. Comparison Between the FE and MSG Approaches

**Figure 9:**
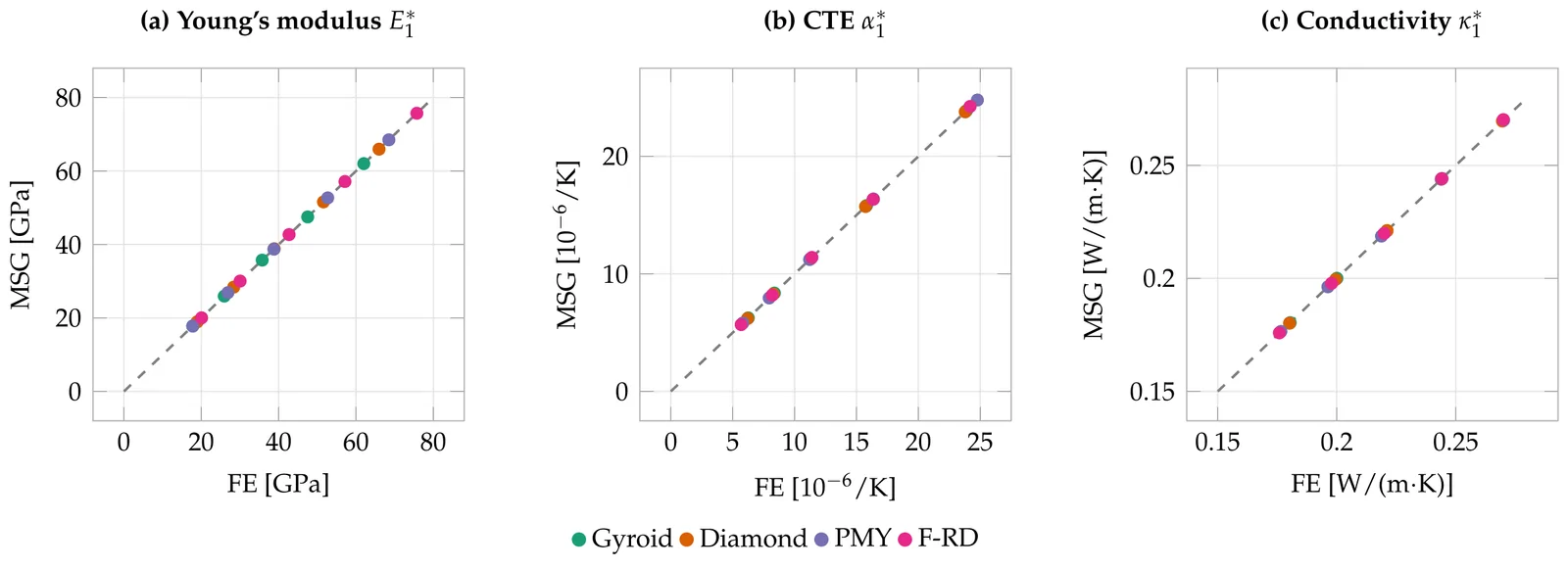
Parity plots comparing FE- and MSG-predicted effective properties across all four topologies and five TPMS-phase volume fractions: (**a**) Young’s modulus $E_{1}^{*}$, (**b**) CTE $\alpha_{1}^{*}$, (**c**) thermal conductivity $\kappa_{1}^{*}$. The dashed line indicates perfect agreement (y=x).

Figure 9 presents parity plots comparing the FE- and MSG-predicted effective Young’s modulus, CTE, and thermal conductivity for all four topologies and all five TPMS-phase volume fractions considered.

All data points lie close to the y=x line for all topologies, TPMS-phase volume fractions and properties. This confirms the close agreement between the two approaches, which was expected on theoretical grounds (Section 2.5). Table 11 summarizes the relative error between the MSG and FE predictions, |MSG−FE|/FE×100%, for the representative axial elastic modulus, shear modulus, Poisson’s ratio, CTE, and thermal conductivity. Values are maximized over the five TPMS-phase volume fractions for each topology.

The maximum relative error between the two approaches is below 0.3% for all five quantities in Table 11, across all four topologies and the full volume-fraction range investigated. The gyroid and diamond topologies agree to within 0.02%. The largest values are found for F-RD (up to 0.27%, for the CTE) and PMY (up to 0.14%, for the Young’s modulus). Both methods solve the same homogenization problem on the same mesh. A difference of this size therefore reflects only numerical details, such as element integration and solver tolerances. It has no bearing on the property trends discussed above. The mesh-refinement sequences of Table 4 confirm that the difference does not grow with refinement. Since the FE and MSG analyses of all twenty configurations use the same meshes, the remaining differences are attributable to the two solution procedures and not to the discretization.

### 3.9. Computational Cost

The computational cost is first characterized by two quantities. They do not depend on the hardware or on the settings of the linear solver. They are the size of the linear systems and the number of matrix decompositions. Table 12 gives, for ten configurations, the number of equations solved by Abaqus and the number of degrees of freedom solved by CmbsFE. For Abaqus, this is the number of equations reported in its message file, after elimination of the periodic constraint equations. It is not the total number of variables of the model, which includes the eliminated degrees of freedom. On identical meshes, the two approaches solve linear systems of the same size. The numbers differ by at most 3, for systems from $10^{4}$ to $1.8\times 10^{6}$ unknowns. The cost of the two approaches therefore differs through the number of matrix decompositions. For a direct solver, this is typically the dominant part of the cost when the number of right-hand sides is small. For the thermo-elastic problem, both approaches solve seven load cases (six unit strains and one temperature change): MSG with one decomposition, the Abaqus analyses with two (Section 2.5). For the thermal-conductivity problem, both approaches solve three load cases: MSG with one decomposition, the Abaqus analyses with three. For the same linear systems, MSG therefore requires one half (thermo-elastic) and one third (conductivity) of the matrix decompositions of the Abaqus analyses.

The solver wall-clock times of the two codes were first compared with one thread each. Table 13 gives the times for five representative configurations. They were run one after the other on the same workstation (Intel Core i9-11900K, 8 cores, 64 GB of RAM, 2133 MHz). For Abaqus, the time is the wall-clock time of the analysis reported in its data file, with the solver restricted to one thread. It excludes the import of the model and the post-processing of the plugin. For CmbsFE, it is the wall-clock time of the complete run (import of the mesh, assembly and solution). For the thermo-elastic problem, the CmbsFE time is 0.62–0.78 times the Abaqus time. For the thermal-conductivity problem, it is 0.29–0.45 times. These ratios are of the same order as the ratios of the numbers of matrix decompositions (one half and one third). The comparison with four threads follows below.

With four threads, the two codes were compared again on the same five configurations and with the same definitions of the times. For Abaqus, the analyses were run with four CPUs. For CmbsFE, the OpenMP-parallel version of MUMPS (version 5.9.0) was used with an OpenMP-parallel BLAS library (OpenBLAS). The number of threads was set to four through the environment variable OMP_NUM_THREADS. MUMPS can compress the factors of the matrix by block low-rank (BLR) approximations [50]. The blocks of the factors that are numerically of low rank are stored as products of two thin matrices, within a prescribed tolerance. This reduces the number of operations and the memory of the factorization, at the price of an approximate factorization. The option is controlled by the MUMPS parameter ICNTL(35), and it is active by default in CmbsFE. Its benefit grows with the size of the system. For the systems considered here, which have up to $3.5\times 10^{5}$ unknowns, the compression may not pay off. Both settings are therefore reported. The effective properties are identical for the two settings, to the eight digits printed by CmbsFE.

Table 14 gives the wall-clock times. For the thermo-elastic problem, the Abaqus times with four threads are 2.0 to 3.2 times shorter than with one thread. The CmbsFE times are about two times shorter for the quadratic meshes, and 1.4 to 1.7 times shorter for the linear meshes. The CmbsFE time is then 0.96–1.22 times the Abaqus time with BLR on, and 0.94–1.13 times with BLR off. The two codes are therefore comparable, and BLR off is faster by up to 9% for the large quadratic meshes. For the thermal-conductivity problem, the CmbsFE time is 0.42–0.75 times the Abaqus time for both settings.

Table 15 gives the peak memory. Abaqus does not report a measured peak memory. The working set of the solver process was therefore sampled every 0.2 s during the analyses. For CmbsFE, the peak memory of the whole process is reported by the code. For the thermo-elastic problem on the quadratic meshes, CmbsFE needs 1.7 to 1.9 times (BLR on) and 1.6 to 1.8 times (BLR off) the memory of Abaqus. BLR off reduces the peak memory by up to 11% for the gyroid meshes. For the thermal-conductivity problem, the ratios range from 1.1 to 1.3 on the quadratic meshes, and the memory of CmbsFE is lower on the linear meshes of the gyroid and the F-RD. The memory requirement of CmbsFE is thus higher than that of Abaqus for the thermo-elastic problem, whereas its solution time is similar. The linear-solver settings of CmbsFE have not been tuned for memory.

## 4. Conclusions

This study has compared FE-based and MSG-based homogenization for predicting the effective elastic properties, coefficients of thermal expansion (CTEs), and thermal conductivities of TPMS-based interpenetrating phase composites (IPCs). Four topologies (gyroid, diamond, PMY, and F-rhombic dodecahedron (F-RD)) were investigated over TPMS-phase volume fractions from 0.1 to 0.5. Conforming-mesh RUC models were analyzed identically by both approaches.

Both frameworks were verified against published literature results for single-phase TPMS lattices, with predictions agreeing to within 1.9%. This confirms that the homogenization procedures developed here reproduce established elastic results before being extended to the new two-phase IPC configurations. All four IPC topologies exhibit a consistent multifunctional trade-off with TPMS-phase volume fraction. Increasing TPMS-phase volume fraction raises stiffness. It also reduces both CTE and thermal conductivity. All three properties are governed by the same competition between the stiff, low-CTE, low-conductivity TPMS phase and the compliant, high-CTE, higher-conductivity matrix phase. The F-RD topology is the stiffest of the four at every TPMS-phase volume fraction studied. It has the lowest CTE and the lowest conductivity only at the highest TPMS-phase volume fraction, ρ=0.5. There it is the clear multifunctional choice for the constituent properties considered here (Table 1). In this material pair, the stiffer TPMS phase also has the lower CTE and the lower conductivity. The monotonic trends with volume fraction are therefore partly built into the constituent contrasts (modulus ratio 20, CTE ratio 55, conductivity ratio 3). The topology ranking should not be generalized to other material combinations without further analysis. At lower TPMS-phase volume fractions, diamond and PMY offer lower CTE and conductivity than F-RD, even though F-RD remains stiffer. Topology selection therefore depends on both the target TPMS-phase volume fraction and the property being prioritized. The effective elastic response is cubic but not isotropic (Zener ratio between 0.83 and 1.10, F-RD being the most anisotropic), whereas the effective CTE and conductivity tensors are isotropic within discretization error. The effective properties of the F-RD topology at ρ=0.1 require a level-set sampling of at least 40 to reproduce the target volume fraction. MSG predictions agree with FE-RUC to within 0.3% for the elastic properties, the CTE and the thermal conductivity, across all topologies and volume fractions. In terms of computational cost, the linear systems solved by the two approaches on identical meshes have the same size, whereas MSG requires one matrix decomposition per physics, against two (thermo-elastic) and three (conductivity) for the Abaqus analyses (Table 12). With one thread for both codes, the solver time of CmbsFE was 0.6–0.8 times (thermo-elastic) and 0.3–0.5 times (conductivity) that of Abaqus (Table 13). With four threads, it was 0.9–1.2 times (thermo-elastic) and 0.4–0.8 times (conductivity) that of Abaqus (Table 14), and its peak memory was 1.7–1.9 times that of Abaqus for the thermo-elastic problem on the quadratic meshes (Table 15). This level of agreement supports the use of MSG as an accurate alternative to FE-RUC homogenization in multifunctional design studies of TPMS-based IPCs.

Future work could extend this framework to additional TPMS topologies and volume fractions. It could incorporate nonlinear or temperature-dependent constituent behavior. It should also validate experimentally the predicted thermo-elastic and transport properties of the two-phase IPCs. Such a validation could use specimens made by multi-material additive manufacturing or by infiltration of a printed TPMS network. The elastic constants could be measured by uniaxial or compression tests combined with ultrasonic or resonant methods. The CTE could be measured by dilatometry or thermomechanical analysis. The thermal conductivity could be measured by the laser-flash, hot-disk or guarded hot-plate methods. Finally, the present models assume perfectly bonded phases. FE-RUC can account for contact, sliding or debonding at the matrix–TPMS interface. This is an advantage of FE over the MSG formulation used here. Linear imperfect-interface models have been incorporated in micromechanics approaches by our group [31,32]. A general contact capability is not available in the present MSG implementation.

## Figures and Tables

**Table 1:** Constituent phase properties used in the numerical models, after [39,46] (elastic properties and CTEs) and representative values assigned for this study (thermal conductivity and volumetric heat capacity, Section 2.2).

Phase	*E* [GPa]	ν [–]	α [$10^{-6}$ 1/K]	κ [W/(m·K)]	$c_{v}$ [MJ/(m^3^·K)]
Matrix phase	10	0.30	44.0	0.30	1.65
TPMS phase	200	0.30	0.8	0.10	1.65

**Table 2:** Mesh convergence study for the gyroid TPMS-based IPC at TPMS-phase volume fraction ρ=0.1 (FE analysis).

$l_{e}$ [mm]	No. of Elements	$E_{1}^{*}$ [GPa]	$\alpha_{1}^{*}$ [$10^{-6}$/K]	$\kappa_{1}^{*}$ [W/(m·K)]
10.0	75,839	17.797	23.891	0.270
7.0	80,020	17.797	23.891	0.270
6.0 ^1^	94,314	17.796	23.892	0.270
5.0	151,667	17.793	23.895	0.270
4.5	198,182	17.791	23.897	0.270
4.0	320,970	17.790	23.898	0.270
3.0	465,576	17.788	23.899	0.270

^1^ Mesh density adopted for the RUCs analyzed in this study.

**Table 3:** Mesh convergence study for the gyroid TPMS-based IPC at TPMS-phase volume fraction ρ=0.5 (FE analysis).

$l_{e}$ [mm]	No. of Elements	$E_{1}^{*}$ [GPa]	$\alpha_{1}^{*}$ [$10^{-6}$/K]	$\kappa_{1}^{*}$ [W/(m·K)]
10.0	72,358	62.064	6.253	0.180
7.0	75,204	62.055	6.253	0.180
6.0 ^1^	88,095	62.020	6.255	0.180
5.0	163,975	61.974	6.257	0.180
4.5	227,672	61.958	6.257	0.180
4.0	364,217	61.943	6.258	0.180
3.0	452,675	61.938	6.259	0.180

^1^ Mesh density adopted for the RUCs analyzed in this study.

**Table 4:** FE and MSG predictions on identical quadratic-tetrahedron meshes for the gyroid and PMY IPCs at ρ=0.5 (level-set sampling 25). Δ=(MSG−FE)/FE×100%.

Topology	$l_{e}$ [mm]	No. of Elements	$E_{1}^{*}$ [GPa]			$\alpha_{1}^{*}$ [$10^{-6}$/K]			$\kappa_{1}^{*}$ [W/(m·K)]		
			FE	MSG	Δ [%]	FE	MSG	Δ [%]	FE	MSG	Δ [%]
Gyroid	10.0	72,358	62.064	62.032	−0.052	6.253	6.256	+0.048	0.180	0.180	0.000
	6.0	88,095	62.020	62.008	−0.019	6.255	6.255	0.000	0.180	0.180	0.000
	4.5	227,672	61.958	61.954	−0.006	6.257	6.258	+0.016	0.180	0.180	0.000
	3.0	452,675	61.938	61.935	−0.005	6.259	6.259	0.000	0.180	0.180	0.000
PMY	9.0	81,024	68.615	68.615	0.000	5.814	5.814	0.000	0.177	0.177	0.000
	6.0	109,819	68.503	68.496	−0.010	5.820	5.820	0.000	0.177	0.177	0.000
	4.0	394,117	68.326	68.326	0.000	5.830	5.830	0.000	0.176	0.176	0.000

**Table 5:** Sensitivity to the level-set sampling resolution for the F-RD IPC at ρ=0.1 (linear tetrahedra, $l_{e}=6$ mm). $V_{1}/V$ is the TPMS volume fraction of the generated mesh, to be compared with the target value 0.1.

Sampling	No. of Elements	$V_{1}/V$ [–]	$E_{1}^{*}$ [GPa]		$\alpha_{1}^{*}$ [$10^{-6}$/K]		$\kappa_{1}^{*}$ [W/(m·K)]	
			FE	MSG	FE	MSG	FE	MSG
25	66,899	0.4872	57.566	57.566	7.061	7.061	0.186	0.186
40	258,088	0.1001	20.076	20.032	24.180	24.247	0.270	0.270
60	655,051	0.1000	19.862	19.862	24.313	24.313	0.270	0.270

**Table 6:** Verification of the FE and MSG frameworks against literature results for single-phase TPMS lattices.

Topology	Source	ρ	$E_{1}^{*}$ [GPa] (% Error) ^1^	$G_{12}^{*}$ [GPa]	$\nu_{12}^{*}$ [–]
Gyroid	Refai et al. [19]	0.1	3.496 (–)	1.415	0.341
	This study, FE	0.1	3.561 (+1.86)	1.435	0.341
	This study, MSG	0.1	3.548 (+1.49)	1.430	0.340
Diamond	Zhang et al. [21], FEM	0.3	26.79 (–)	–	–
	Zhang et al. [21], EXP 1	0.3	25.65 (–)	–	–
	Zhang et al. [21], EXP 2	0.3	26.79 (–)	–	–
	This study, FE	0.3	26.81 (+0.08)	8.754	0.271
	This study, MSG	0.3	26.79 (+0.01)	8.746	0.271

^1^ Relative to the corresponding FEM literature value (Zhang et al. EXP 2 coincides with their FEM prediction to the precision reported); the FE prediction deviates from Zhang et al.’s EXP 1 measurement by +4.53%.

**Table 7:** Effective elastic properties predicted by FE and MSG for the four TPMS-based IPC topologies, underlying Figure 5.

Topology	ρ [–]	$E_{1}^{*}$ [GPa]		$G_{12}^{*}$ [GPa]		$\nu_{12}^{*}$ [–]	
		FE	MSG	FE	MSG	FE	MSG
Gyroid	0.1	17.80	17.80	7.18	7.18	0.301	0.301
	0.2	25.95	25.95	10.69	10.69	0.303	0.303
	0.3	35.75	35.75	15.00	15.00	0.299	0.299
	0.4	47.51	47.51	20.22	20.22	0.294	0.294
	0.5	62.02	62.01	26.52	26.52	0.289	0.289
Diamond	0.1	18.94	18.94	6.94	6.94	0.290	0.290
	0.2	28.40	28.40	10.16	10.16	0.285	0.285
	0.3	38.86	38.86	14.27	14.27	0.282	0.282
	0.4	51.60	51.60	19.66	19.66	0.278	0.278
	0.5	65.96	65.96	26.05	26.05	0.277	0.277
PMY	0.1	17.80	17.80	7.32	7.33	0.295	0.294
	0.2	26.89	26.89	11.30	11.30	0.289	0.289
	0.3	38.78	38.73	16.23	16.21	0.284	0.283
	0.4	52.70	52.70	21.89	21.89	0.282	0.282
	0.5	68.50	68.50	28.26	28.26	0.280	0.280
F-RD	0.1	20.08	20.04	6.98	6.96	0.273	0.273
	0.2	30.05	30.05	9.80	9.80	0.265	0.265
	0.3	42.70	42.70	14.24	14.24	0.258	0.258
	0.4	57.15	57.15	19.71	19.71	0.257	0.257
	0.5	75.74	75.74	26.83	26.83	0.262	0.262

**Table 8:** Zener anisotropy ratio $A=2C_{44}/\left(C_{11}-C_{12}\right)$ of the effective elasticity tensor (MSG) as a function of the TPMS volume fraction ρ.

Topology	ρ=0.1	ρ=0.2	ρ=0.3	ρ=0.4	ρ=0.5
Gyroid	1.050	1.073	1.091	1.102	1.103
Diamond	0.945	0.920	0.942	0.974	1.009
PMY	1.065	1.083	1.074	1.065	1.056
F-RD	0.885	0.825	0.839	0.867	0.894

**Table 9:** Effective axial coefficient of thermal expansion $\alpha_{1}^{*}$ predicted by FE and MSG for the four TPMS-based IPC topologies, underlying Figure 6.

Topology	ρ [–]	$\alpha_{1}^{*}$ FE [$10^{-6}$/K]	$\alpha_{1}^{*}$ MSG [$10^{-6}$/K]
Gyroid	0.1	23.892	23.892
	0.2	15.811	15.811
	0.3	11.286	11.286
	0.4	8.369	8.369
	0.5	6.255	6.254
Diamond	0.1	23.787	23.787
	0.2	15.736	15.736
	0.3	11.297	11.297
	0.4	8.289	8.289
	0.5	6.203	6.203
PMY	0.1	24.769	24.791
	0.2	16.373	16.373
	0.3	11.216	11.225
	0.4	7.952	7.952
	0.5	5.820	5.820
F-RD	0.1	24.181	24.246
	0.2	16.345	16.345
	0.3	11.401	11.401
	0.4	8.194	8.194
	0.5	5.684	5.684

**Table 10:** Effective axial thermal conductivity $\kappa_{1}^{*}$ predicted by FE and MSG for the four TPMS-based IPC topologies, underlying Figure 7.

Topology	ρ [–]	$\kappa_{1}^{*}$ FE [W/(m·K)]	$\kappa_{1}^{*}$ MSG [W/(m·K)]
Gyroid	0.1	0.26990	0.26990
	0.2	0.24421	0.24421
	0.3	0.22103	0.22103
	0.4	0.20007	0.20007
	0.5	0.18045	0.18042
Diamond	0.1	0.26962	0.26962
	0.2	0.24389	0.24389
	0.3	0.22115	0.22115
	0.4	0.19957	0.19957
	0.5	0.18017	0.18017
PMY	0.1	0.27013	0.27007
	0.2	0.24395	0.24395
	0.3	0.21881	0.21873
	0.4	0.19629	0.19629
	0.5	0.17654	0.17654
F-RD	0.1	0.26999	0.27026
	0.2	0.24418	0.24418
	0.3	0.21992	0.21992
	0.4	0.19785	0.19785
	0.5	0.17591	0.17591

**Table 11:** Maximum relative error between MSG and FE predictions over ρ=0.1–0.5, by topology.

Topology	$E_{1}^{*}$ [%]	$G_{12}^{*}$ [%]	$\nu_{12}^{*}$ [%]	$\alpha_{1}^{*}$ [%]	$\kappa_{1}^{*}$ [%]
Gyroid	0.01	0.02	0.02	0.01	0.02
Diamond	0.00	0.00	0.00	0.00	0.00
PMY	0.14	0.12	0.08	0.09	0.03
F-RD	0.20	0.15	0.13	0.27	0.10

Maximum absolute value of |MSG−FE|/FE×100% over the five TPMS-phase volume fractions ρ=0.1–0.5 investigated for each topology.

**Table 12:** Size of the linear systems: number of equations solved by Abaqus and number of degrees of freedom solved by CmbsFE, for the thermo-elastic and the thermal-conductivity analyses. Gyroid and PMY at ρ=0.5, F-RD at ρ=0.1 (linear elements); quadratic elements otherwise ($l_{e}$ in mm).

Topology	Mesh	No. of Elements	Thermo-Elastic		Conductivity	
			Abaqus	CmbsFE	Abaqus	CmbsFE
Gyroid	$l_{e}=10$	72,358	288,390	288,387	96,131	96,129
Gyroid	$l_{e}=6$	88,095	350,655	350,652	116,886	116,884
Gyroid	$l_{e}=4.5$	227,672	903,762	903,759	301,255	301,253
Gyroid	$l_{e}=3$	452,675	1,796,583	1,796,580	598,862	598,860
PMY	$l_{e}=9$	81,024	323,010	323,007	107,671	107,669
PMY	$l_{e}=6$	109,819	436,641	436,638	145,548	145,546
PMY	$l_{e}=4$	394,117	1,565,931	1,565,930	521,978	521,976
F-RD	res. 25	66,899	32,688	32,685	10,897	10,895
F-RD	res. 40	258,088	124,659	124,656	41,554	41,552
F-RD	res. 60	655,051	317,547	317,544	105,850	105,848

**Table 13:** Solver wall-clock time [s] of Abaqus and CmbsFE, with one thread each, on the same workstation (Intel Core i9-11900K, 8 cores, 64 GB of RAM, 2133 MHz). Gyroid and PMY at ρ=0.5, F-RD at ρ=0.1; quadratic elements unless stated ($l_{e}$ in mm). The ratio is the CmbsFE time divided by the Abaqus time.

Topology	Mesh	Abaqus [s]	CmbsFE [s]	Ratio [–]
Thermo-elastic problem				
Gyroid	$l_{e}=10$	183	129	0.71
Gyroid	$l_{e}=6$	272	190	0.70
Gyroid	$l_{e}=6$, linear	10	6	0.62
PMY	$l_{e}=9$	199	155	0.78
F-RD	res. 40, linear	46	30	0.64
Thermal-conductivity problem				
Gyroid	$l_{e}=10$	29	11	0.39
Gyroid	$l_{e}=6$	37	17	0.45
Gyroid	$l_{e}=6$, linear	7	2	0.29
PMY	$l_{e}=9$	31	13	0.41
F-RD	res. 40, linear	20	7	0.33

**Table 14:** Solver wall-clock time [s] with four threads: Abaqus (four CPUs) and CmbsFE (OpenMP MUMPS, OMP_NUM_THREADS=4), with block low-rank compression (BLR) on and off, on the same workstation as Table 13. Same configurations as in Table 13. The ratio is the CmbsFE time divided by the Abaqus time.

Topology	Mesh	Abaqus [s]	BLR on		BLR off	
			CmbsFE [s]	Ratio [–]	CmbsFE [s]	Ratio [–]
Thermo-elastic problem						
Gyroid	$l_{e}=10$	58	65.4	1.13	60.9	1.05
Gyroid	$l_{e}=6$	86	95.0	1.10	86.4	1.00
Gyroid	$l_{e}=6$, linear	5	4.8	0.96	4.7	0.94
PMY	$l_{e}=9$	65	79.0	1.22	73.5	1.13
F-RD	res. 40, linear	17	18.7	1.10	18.3	1.08
Thermal-conductivity problem						
Gyroid	$l_{e}=10$	12	8.6	0.72	8.3	0.69
Gyroid	$l_{e}=6$	15	11.3	0.75	10.9	0.73
Gyroid	$l_{e}=6$, linear	4	1.7	0.42	1.7	0.42
PMY	$l_{e}=9$	13	9.7	0.75	9.5	0.73
F-RD	res. 40, linear	9	5.3	0.59	5.2	0.58

**Table 15:** Peak memory [GB] with four threads: peak working set of the solver process of Abaqus (four CPUs) and peak memory of the CmbsFE process (OpenMP MUMPS, four threads), with BLR on and off. Same configurations as in Table 13. The ratio is the CmbsFE value divided by the Abaqus value.

Topology	Mesh	Abaqus [GB]	BLR on		BLR off	
			CmbsFE [GB]	Ratio [–]	CmbsFE [GB]	Ratio [–]
Thermo-elastic problem						
Gyroid	$l_{e}=10$	5.51	9.80	1.78	8.68	1.58
Gyroid	$l_{e}=6$	7.21	12.64	1.75	11.44	1.59
Gyroid	$l_{e}=6$, linear	0.71	0.85	1.20	0.84	1.18
PMY	$l_{e}=9$	6.00	11.07	1.85	11.04	1.84
F-RD	res. 40, linear	2.06	3.14	1.52	2.89	1.40
Thermal-conductivity problem						
Gyroid	$l_{e}=10$	1.05	1.29	1.22	1.19	1.13
Gyroid	$l_{e}=6$	1.28	1.70	1.33	1.56	1.22
Gyroid	$l_{e}=6$, linear	0.39	0.21	0.55	0.20	0.52
PMY	$l_{e}=9$	1.14	1.46	1.28	1.34	1.17
F-RD	res. 40, linear	0.78	0.63	0.81	0.59	0.76

## Data Availability

The original contributions presented in this study are included in the article. Further inquiries can be directed to the corresponding author.

## Abbreviations

The following abbreviations are used in this manuscript:

CAD	Computer-aided design
CTE	Coefficient of thermal expansion
DOF	Degree of freedom
FE	Finite element
F-RD	F-rhombic dodecahedron (Schoen F-RD surface)
IPC	Interpenetrating phase composite
MSG	Mechanics of structure genome
PBC	Periodic boundary condition
PMY	PMY triply periodic minimal surface
RUC	Representative unit cell
SG	Structure genome
TPMS	Triply periodic minimal surface
VAMUCH	Variational Asymptotic Method for Unit Cell Homogenization
